# AMPK in skeletal muscle function and metabolism

**DOI:** 10.1096/fj.201700442R

**Published:** 2018-01-05

**Authors:** Rasmus Kjøbsted, Janne R. Hingst, Joachim Fentz, Marc Foretz, Maria-Nieves Sanz, Christian Pehmøller, Michael Shum, André Marette, Remi Mounier, Jonas T. Treebak, Jørgen F. P. Wojtaszewski, Benoit Viollet, Louise Lantier

**Affiliations:** * Section of Molecular Physiology, Department of Nutrition, Exercise, and Sports, Faculty of Science, University of Copenhagen, Copenhagen, Denmark;; † INSERM, Unité 1016, Institut Cochin, Paris, France;; ‡ Centre National de la Recherche Scientifique (CNRS), Unité Mixte de Recherche (UMR) 8104, Paris, France;; § Université Paris Descartes, Sorbonne Paris Cité, Paris, France;; ¶ Department of Cardiovascular Surgery, Inselspital, Bern University Hospital, University of Bern, Bern, Switzerland, and; ‖ Department of Biomedical Research, University of Bern, Bern, Switzerland;; # Internal Medicine Research Unit, Pfizer Global Research and Development, Cambridge, Massachusetts, USA;; ** Axe Cardiologie, Quebec Heart and Lung Research Institute, Laval University, Québec, Canada;; †† Institute for Nutrition and Functional Foods, Laval University, Québec, Canada;; ‡‡ Institute NeuroMyoGène, Université Claude Bernard Lyon 1, INSERM Unité 1217, CNRS UMR, Villeurbanne, France;; §§ Section of Integrative Physiology, Novo Nordisk Foundation Center for Basic Metabolic Research, Faculty of Health and Medical Sciences, University of Copenhagen, Copenhagen, Denmark;; ¶¶ Department of Molecular Physiology and Biophysics, Vanderbilt University, Nashville, Tennessee, USA;; ‖‖ Mouse Metabolic Phenotyping Center, Vanderbilt University, Nashville, Tennessee, USA

**Keywords:** exercise, mitochondria, glucose uptake, plasticity, diabetes

## Abstract

Skeletal muscle possesses a remarkable ability to adapt to various physiologic conditions. AMPK is a sensor of intracellular energy status that maintains energy stores by fine-tuning anabolic and catabolic pathways. AMPK’s role as an energy sensor is particularly critical in tissues displaying highly changeable energy turnover. Due to the drastic changes in energy demand that occur between the resting and exercising state, skeletal muscle is one such tissue. Here, we review the complex regulation of AMPK in skeletal muscle and its consequences on metabolism (*e.g.*, substrate uptake, oxidation, and storage as well as mitochondrial function of skeletal muscle fibers). We focus on the role of AMPK in skeletal muscle during exercise and in exercise recovery. We also address adaptations to exercise training, including skeletal muscle plasticity, highlighting novel concepts and future perspectives that need to be investigated. Furthermore, we discuss the possible role of AMPK as a therapeutic target as well as different AMPK activators and their potential for future drug development.—Kjøbsted, R., Hingst, J. R., Fentz, J., Foretz, M., Sanz, M.-N., Pehmøller, C., Shum, M., Marette, A., Mounier, R., Treebak, J. T., Wojtaszewski, J. F. P., Viollet, B., Lantier, L. AMPK in skeletal muscle function and metabolism.

One fundamental function of skeletal muscle is to generate mechanical force to support body posture and to facilitate a wide variety of movements. Besides this role in body motility, skeletal muscle has been shown to be important for regulating whole-body metabolism. Skeletal muscle demonstrates high malleability and can adapt its contractile composition and metabolic properties in response to a number of physiologic conditions, including exercise. Such adaptations are reflected by changes in muscle size, fiber type distribution, contractile velocity, force production, and endurance capacity, being the result of the functional demands of the contractile activity (1, 2). This plasticity may involve short- and long-term mechanisms, leading to changes in protein abundance and activity (1–3). These changes are mediated by activation and repression of specific intracellular signaling events that govern effectors involved in metabolic pathways and transcription/translation processes of exercise-responsive genes (4). The intracellular signaling mechanisms that modify skeletal muscle function in response to exercise are regulated by perturbations in muscle cell homeostasis, including alterations in tissue perfusion, oxygen tension, redox state, calcium (Ca^2+^) dynamics, and ATP turnover (5). Evidence suggests that ATP turnover in skeletal muscle may increase by >100-fold in response to exercise (6). Keeping cellular ATP concentrations fairly constant during such conditions represents a major challenge to the cell and highlights the vast dynamics of muscle energy metabolism. Because skeletal muscle ATP consumption increases during exercise, intracellular AMP concentrations may accumulate as a result of the adenylate kinase reaction. This increases cellular AMP/ATP and ADP/ATP ratios, leading to activation of AMPK (7). This kinase is considered a central sensor of intracellular energy status and maintains energy stores by regulating anabolic and catabolic pathways, thereby ensuring a balance between energy supply and demand (8). In skeletal muscle, acute pharmacological activation of AMPK has been shown to promote glucose transport and fatty acid oxidation (9) while suppressing glycogen synthase activity and protein synthesis (10, 11). In addition, chronic activation of AMPK reduces markers of skeletal muscle fragility (12) and enhances muscle fiber oxidative capacity by stimulating mitochondrial biogenesis (13–15). These events are initiated by AMPK downstream phosphorylation of key metabolic enzymes as well as transcription factors that modulate cellular metabolism in order to handle both current and future metabolic challenges. Several excellent reviews have examined the role of AMPK in regulating skeletal muscle function and metabolism (16–49). Therefore, this review addresses novel concepts and future perspectives of AMPK in skeletal muscle that need to be experimentally validated and tested.

## AMPK STRUCTURE AND EXPRESSION

**Figure 1.**
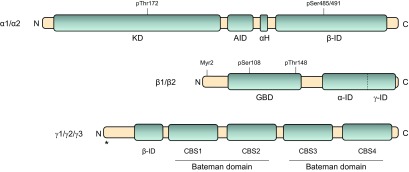
Structure of mammalian AMPK subunits. AMPK is a heterotrimeric protein consisting of 1 catalytic subunit (α subunit) and 2 regulatory subunits (β and γ subunit). The α subunit contains a kinase domain (KD), the activity of which relies on the phosphorylation of Thr172 by upstream AMPK kinases. The KD is followed by an autoinhibitory domain (AID) and an α-hook (αH), which seem to be important for AMP-regulated catalytic activity. At the α-C terminus, a β-interacting domain (β-ID) has been detected that binds to the C-terminal domain of the β subunit. Phosphorylation of Ser485/491 in the β-ID during insulin stimulation has been suggested to regulate kinase activity. The β subunit is subjected to myristoylation at the N terminus, which enhances phosphorylation of α-Thr172 by AMP/ADP and facilitates AMPK translocation to specific intracellular compartments. At the center, the β subunit contains a GBD that causes AMPK to bind to glycogen particles. Within the GBD, 2 phosphorylation sites have been found (Ser108 and Thr148) that seem to regulate binding capacity to glycogen particles as well as kinase activity. An α- and γ-interaction domain (α-ID, γ-ID) is located at the β-C terminus that acts as a scaffold keeping the heterotrimeric complex together. The γ-subunit contains 4 cystathionine–β-synthase (CBS) domains. These occur in tandem pairs, also known as Bateman domains, and are involved in adenosine nucleotide binding. A β-ID is located close to the γ-N terminus. An asterisk denotes that the 3 γ isoforms contain different N-terminal extensions.

AMPK is a heterotrimeric protein complex that consists of a catalytic subunit (α) and 2 regulatory subunits (β and γ), of which several isoforms have been found (α1, α2, β1, β2, γ1, γ2, and γ3) (50) (**Fig. 1**). The α subunit contains the kinase domain, activity of which is highly dependent on the reversible phosphorylation of α-Thr172 (51–53). The β subunit acts as a scaffold for binding the α and γ subunits (54) and contains a glycogen-binding domain (GBD) that likely targets the heterotrimeric complex to glycogen particles (55, 56). The γ subunit functions as a sensor of intracellular energy status through its direct binding of adenosine nucleotides (57). Besides these well-established functions, all 3 subunits contain different domains or posttranslational modifications that may locate AMPK to distinct subcellular compartments. In the α subunit, a nuclear export sequence has been found (58), and myristoylation of the β subunit has been suggested to facilitate AMPK translocation to perinuclear speckles and mitochondrial membranes (59–61). The γ subunit isoforms differ in their N-terminal extensions, which also appear to determine AMPK localization (62–64). Thus, in skeletal muscle fibers, the γ1 isoform is localized to the *z* disk, whereas the γ3 isoform is found in the nucleus and along the *z* disk and I-band in a pattern that closely resembles the T-tubule/sarcoplasmic reticulum structures (64). The functional role of AMPK in different subcellular locations has not received much attention, but recent findings indicate that it may represent another way of regulating AMPK activity. For example, the unspecific AMPK activator PT-1 appears to only activate γ1 complexes in mouse skeletal muscle, whereas it displays no isoform selectivity in HEK293 cells stably expressing each of the 3 γ isoforms (65). Because PT-1 activates AMPK by inhibiting the mitochondrial respiratory chain (65), this may signify an important role of the γ1-associated complexes in monitoring ATP synthesis, and the highly contraction-responsive γ3-containing complex may serve as the major sensor of ATP consumption in skeletal muscle. On the other hand, the PT-1 concentration previously used to stimulate isolated skeletal muscle (65) may have been insufficient to activate γ3-containing complexes. Recent evidence from cell-based studies also indicates that AMPK subunit composition influences sensitivity to AMP, which likely contributes to the specialized functions of AMPK heterotrimeric subtypes (57, 66–68).

The 7 different AMPK subunit isoforms give rise to 12 heterotrimeric combinations that seem to be expressed in a tissue-specific manner. Thus, in skeletal muscle preparations from human and mouse, all subunit isoforms have been detected, but only a subset of possible heterotrimeric complexes seems to exist (69, 70). In human skeletal muscle (vastus lateralis), 3 different complexes have been described (α2β2γ1, α2β2γ3, and α1β2γ1) (69), whereas 5 complexes have been identified in mouse skeletal muscle (α2β2γ1, α2β2γ3, α2β1γ1, α1β2γ1, and α1β1γ1) (70) (**Table 1**). In addition, some AMPK subunits are expressed in a fiber type–dependent manner (71), which may explain the relative distribution of complexes between different muscles (70). Currently, it is not known why mouse skeletal muscle appears to express two additional complexes (β1-associated) compared with human skeletal muscle. Based on findings in muscle-specific AMPKα and AMPKβ double-knockout (KO) mice (AMPKα mdKO and AMPK β1β2M-KO mice, respectively) (72–74), the majority of β1-associated complexes detected in a crude sample of skeletal muscle seem to derive from nonmuscle tissue (*e.g.*, connective tissue, neuronal cells, adipocytes, endothelial cells, etc.). This is in line with the notion that the α1β1γ1 complex is the most ubiquitously expressed complex of AMPK (68, 75). Interestingly, the β1 subunit is also detected in sample preparations of human skeletal muscle, but, in light of findings from coimmunoprecipitations of AMPKα2, -α1, -γ1, and -γ3, it does not seem to engage in stable complex formation or contribute to any measurable AMPK activity (69, 76). Although it is generally thought that all 3 AMPK subunits must be present to form a stable complex, it has been demonstrated in cell models that stable β1γ1 complexes can form in the absence of catalytic subunits (77). Whether formation of βγ heterodimer complexes also occurs in mature skeletal muscle may be derived from observations in two muscle-specific AMPK double-KO mouse models. Thus, in the AMPKβ1β2M-KO model it seems evident that expression of β2 protein is restricted to the myocytes (74). Interestingly, significant amounts of β2 protein have been detected in a skeletal muscle sample preparation from the AMPKα mdKO mouse model (73), which may suggest the formation of stable βγ complexes in mature skeletal muscle, assuming that single unbound subunits of AMPK are targeted for degradation. This may also be inferred from the AMPKβ1β2M-KO mouse model, which does not express α2 protein in skeletal muscle (74), indicating that the β subunit is vital for maintaining AMPKα muscle protein expression. Collectively, these observations may suggest that the AMPK heterodimer (βγ) exists in skeletal muscle tissue and raises the possibility of a regulatory mechanism facilitating the association of catalytic subunits with regulatory complexes. Alternatively, and somewhat speculatively, other proteins may bind to the regulatory heterodimer complex to regulate their activity or cellular localization. In this context, 12 protein kinases related to AMPKα1 and AMPKα2 have been detected in the human kinome (78). These are known as AMPK-related kinases, and, with a single exception, these are activated by upstream kinase liver kinase B1 (LKB1) (79). Although green fluorescent protein-transporter associated with antigen processing–tagged versions of these kinases do not appear to bind AMPK β and γ subunits (80), the sucrose nonfermenting AMPK-related kinase (SNARK/NUAK2) is activated in skeletal muscle by 5-amino-1-β-d-ribofuranosyl-imidazole-4-carboxamide (AICAR), contraction, and exercise (81, 82), indicating that SNARK activity is regulated similarly to AMPK. Is it possible that AMPK βγ subunits form heterotrimeric complexes with SNARK, facilitating its regulation by adenine nucleotides? If so, it could be anticipated that AMPKβ-deficient skeletal muscle exhibits a phenotype different from that of AMPKα-deficient skeletal muscle. Indeed, in skeletal muscle several phenotypic differences have been observed between AMPKα mdKO and AMPKβ1β2M-KO mice, including muscle mass, ATP levels, mitochondrial DNA and structure, citrate synthase activity, and peroxisome proliferator–activated receptor γ coactivator 1α (PGC-1α) mRNA levels (72, 74). In light of these observations, it has recently been reported that SNARK may be involved in the maintenance of muscle mass with age (83). Assuming that the potential binding of SNARK to AMPKβγ subunits induces an increase in SNARK activity and/or protects SNARK from degradation and that the association between SNARK and AMPKβγ is enhanced in AMPKα-deprived muscle, this could explain the increase in muscle mass observed in skeletal muscle deprived of AMPKα subunits (72).

**TABLE 1.** Relative distribution and basal activity of AMPK heterotrimeric complexes detected in human and mouse skeletal muscle

Trimer complex			*Mus musculus*			
	*Homo sapiens* vastus lateralis		Extensor digitorum longus		Soleus	
	Relative expression	Relative basal activity	Relative expression	Relative basal activity	Relative expression	Relative basal activity
α2β2γ1	∼65%	∼30%	∼70%	∼50%	∼60%	∼35%
α2β2γ3	∼20%	<5%	∼20%	∼20%	<2%	<2%
α1β2γ1	∼15%	∼65%	<5%	∼25%	∼20%	∼50%
α2β1γ1	N.D.	N.D.	<3%	<5%	∼10%	<15%
α1β1γ1	N.D.	N.D.	<2%		<8%	

The composition of AMPK heterotrimeric complexes was estimated from immunoprecipitation experiments in extensor digitorum longus and soleus from C57BL/6 mice as well as human male vastus lateralis. Values adapted from references 70, 76, and 487. N.D., nondetectable.

## REGULATION AND ACTIVATION OF AMPK IN SKELETAL MUSCLE

**Figure 2.**
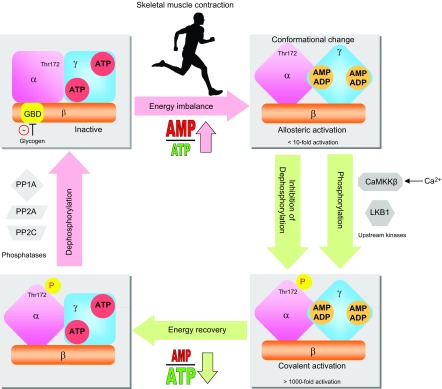
Regulation of AMPK in skeletal muscle during contractile activity. Exercise induces an energy imbalance in muscle, which leads to a rise in intracellular AMP and ADP concentrations. Binding of ADP and AMP at the Bateman domains of the γ subunit causes a conformational change that activates AMPK by up to 10-fold *via* an allosteric mechanism. This conformational change also triggers Thr172 phosphorylation of the α catalytic subunit by the upstream LKB1 and protects against dephosphorylation by protein phosphatases, increasing the activity 100-fold. Together the allosteric effect and α-Thr172 phosphorylation lead to a >1000-fold activation. AMPK is also activated by a rise in the intracellular Ca^2+^ concentration through α-Thr172 phosphorylation catalyzed by CaMKKβ. After exercise and energy repletion, AMPK is converted back to an inactive form by dephosphorylation catalyzed by protein phosphatases (PP1A, PP2A, and PP2C) and undergoes inhibition by glycogen *via* binding to the GBD of the β subunit.

**Figure 3.**
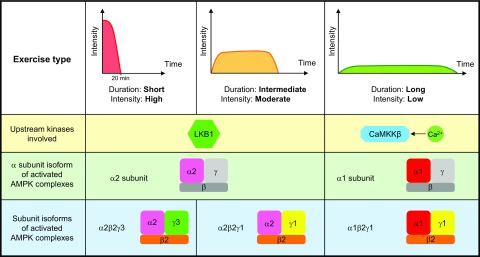
Activation of different AMPK complexes in skeletal muscle is dependent on exercise intensity and duration. In skeletal muscle, LKB1 is the major upstream kinase responsible for the phosphorylation of α2-containing AMPK complexes in response to high/moderate-intensity and short/limited-duration exercise. In contrast, CaMKKβ phosphorylates and activates α1-containing AMPK complexes during long-term exercise at a low intensity. In human vastus lateralis muscle, AMPK activation is restricted to α2β2γ3 heterotrimers during short (up to 20 min) and intense exercise, whereas the α2β2γ1 and α1β2γ1 complexes appear unchanged or even show decreased activation. When exercise is prolonged, α2β2γ1 heterotrimers are activated. During lower-intensity exercise of longer duration, α2β2γ1 and α1β2γ1 complexes are moderately activated. Thus, in skeletal muscle each AMPK heterotrimer combination is regulated in a distinct manner during contraction depending on exercise intensity and duration, which causes a differential functional response.

During skeletal muscle contraction, the adenylate energy charge in muscle is decreased depending on the duration and intensity of exercise (84, 85). As a result, the intracellular AMP/ATP and ADP/ATP ratios increase, which leads to activation of AMPK (7). AMPK activation occurs in two steps: stimulatory allosteric binding of AMP within the γ subunit and covalent activation through reversible phosphorylation on Thr172 in the catalytic α subunit (**Fig. 2**). AMPK activity is stimulated by AMP and ADP and inhibited by ATP binding to the two regulatory Bateman domains of the γ subunit. This competitive binding means that increases in cellular AMP/ATP and ADP/ATP ratios stimulate AMPK allosterically (86–90). The allosteric stimulation has a moderate effect on AMPK activity (<10-fold) (91). More importantly, binding of AMP and/or ADP to the γ subunit induces conformational changes that promote phosphorylation of α-Thr172 (67, 90) and permit protection against dephosphorylation by protein phosphatases PP1, PP2A, and PP2C (41, 92–95). The combined effect of allosteric activation and phosphorylation on α-Thr172 induces a >1000-fold increase in AMPK activity (91). In skeletal muscle, LKB1 is the primary upstream kinase responsible for the phosphorylation of α2-containing AMPK complexes in response to contraction and pharmacological AMPK activators (96–99). To a lesser extent, Ca^2+^/calmodulin-dependent protein kinase kinase β (CaMKKβ) likely phosphorylates and activates AMPKα1 complexes during long-term low-intensity exercise/contraction (100, 101) (**Fig. 3**). LKB1 appears constitutively active (102, 103), whereas CaMKKβ activates AMPK upon an increase in intracellular Ca^2+^ concentrations, even in the absence of adenine nucleotide content imbalance (104, 105). In addition, glycogen has been shown to influence AMPK activity through its interaction with the β subunit. The β subunit contains a GBD that causes AMPK complexes to associate with glycogen particles in cell‐free systems and cultured cells, and this association inhibits AMPK activity (56, 106). This inhibition by glycogen seems to affect mainly α2-containing AMPK complexes (107). Furthermore, *in vivo* studies report an inverse relationship between muscle glycogen content and AMPK activation in rodents (108, 109) and humans (107), although this inhibition by glycogen *in vivo* is not consistently found (110). Interestingly, the β subunit is autophosphorylated at Ser108 and Thr148, which seems to regulate AMPK activity and its binding capacity to glycogen, respectively (59, 111). In addition, findings suggest that insulin reduces AMPK activity in rat skeletal muscle likely through Akt-mediated phosphorylation of Ser485/491 on the α1/α2 subunit (112).

The activation of AMPK in rodent skeletal muscle during exercise was initially described by Winder and Hardie (113), and the first reports of AMPK activation during exercise in human skeletal muscle were published some years later (114–116). Several studies have been carried out to determine the activation profile of the different AMPK complexes during exercise bouts depending on intensity and duration. Typically, AMPK activation is observed only at exercise intensities of a minimum of 60% *V*o_2peak_ (107, 116–119). However, low-intensity exercise at 30–40% of *V*o_2peak_ but performed until exhaustion also activates AMPK in skeletal muscle (119). Moreover, AMPK activation seems dependent on exercise duration (115, 118, 119), although not all studies report this (120). During exercise at high intensity and moderate duration, α2-containing AMPK complexes are predominantly activated (115–118), whereas activity of AMPKα1 complexes have been found to increase, stay unchanged, or decrease in an intensity- and duration-dependent manner (76, 101, 110, 117). Specifically, in human vastus lateralis muscle during a short (up to 20 min) and intense exercise bout, AMPK activation is restricted to γ3-containing complexes (α2β2γ3), whereas activity of the other complexes (α2β2γ1 and α1β2γ1) appears unchanged or even decreased (76). When exercise is prolonged, α2β2γ1 heterotrimers are activated (101). During exercise at lower intensities and of longer duration, a moderate increase in α2β2γ1 activity and a weak increase in α1β2γ1 activity have been observed (101). Thus, in skeletal muscle the different AMPK heterotrimer complexes are regulated in a distinct manner during contraction depending on exercise intensity and duration, which may cause different functional responses. How this differential activation of the specific AMPK complexes is accomplished during exercise needs further investigation. One hypothesis could be that it may in part reflect differences in AMP/ADP sensitivity of the complexes (121). Another possibility could be that the findings relate to regulation and/or recruitment of other cells types (*e.g.*, endothelial cells, adipocytes, and macrophages) within the muscle tissue during exercise. Whatever underlies the differential regulation of AMPK trimer activity in skeletal muscle during exercise, it emphasizes the complexity of these observations, which should be accounted for when evaluating downstream signaling of AMPK. Regarding this finding, distinct phosphorylation signatures of downstream targets have been demonstrated for γ1- and γ3-containing complexes in human skeletal muscle in response to exercise (122), supporting differential functions of the various AMPK heterotrimeric complexes. However, studies investigating the importance of the different complexes for various functions in skeletal muscle are somewhat limited. Yet, it is generally known that activating mutations in the AMPKγ1 and AMPKγ3 subunits promote glycogen storage in skeletal (and cardiac) muscle (123–127). Furthermore, the AMPK α2β2γ3 complex, which is predominantly expressed in skeletal muscle, seems important for AMPK-mediated glucose transport, induction of mitochondrial biogenesis, and improvement in muscle insulin sensitivity (125, 128, 129).

Evidence indicates that AMPK activity in skeletal muscle is regulated differently depending on training status. Thus, several studies have shown that activation of skeletal muscle AMPK by acute exercise, performed at the same absolute and relative intensity, is reduced after a period of exercise training (110, 130–133). This may suggest that the working muscle has an increased ability to maintain energy homeostasis after a period of exercise training or that exercise training diminishes the effect of acute exercise on muscle energy stress, as previously indicated (134). Although these explanations seem plausible, other factors may contribute to the lower exercise-induced activation of AMPK in skeletal muscle after exercise training. One such factor could be elevated glycogen levels in trained muscle that may prevent sufficient activation of AMPK in response to acute exercise. Another factor could be the striking decrease in AMPKγ3 protein content (10–60%) observed in skeletal muscle after exercise training (69, 130, 131, 135). Because the AMPK α2β2γ3 complex is the major complex activated in skeletal muscle during exercise, it would indeed be expected that the exercise-induced activation of AMPK is affected by a reduction in AMPKγ3 protein content after exercise training. Although genetic evidence is lacking, activation of the PPARβ pathway is likely the mechanism responsible for lowering the content of AMPKγ3 protein in skeletal muscle (136).

## AMPK ACTIVATORS

**Figure 4.**
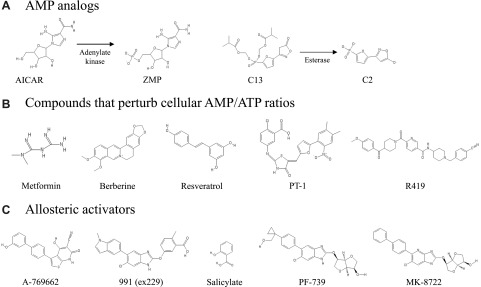
Chemical structure of different AMPK-activating compounds. *A*) Prodrugs that are converted to AMP analogs by enzymes once transported into the cell. *B*) Natural and synthetic compounds that increase intracellular AMP concentrations by disrupting ATP generation in mitochondria. *C*) Direct activators that bind in a pocket between the kinase domain on the α subunit and the carbohydrate-binding module on the β subunit (ADaM site).

Given its pivotal role in metabolism, activation of AMPK has long been thought of as a putative therapeutic target for metabolic disorders such as type 2 diabetes (137). Over the years, a myriad of AMPK activators have emerged in the literature (138). Nevertheless, there are no clinically attractive direct activators of AMPK available to date. This underscores the inherent complexity of activating the AMPK complex. The activators described in the literature can largely be divided into 3 subgroups based on their mechanisms of activation: AMP analogs, compounds that perturb the cellular AMP/ATP ratio, and allosteric activators (**Fig. 4**).

### AMP analogs

The first and, to date, most studied compound in the category of AMP analogs is AICAR (139). AICAR is a prodrug and an adenosine analog that, once transported into the cell by adenosine transporters, is phosphorylated by adenosine kinase into 5-aminoimidazole-4-carboxamide ribonucleotide (ZMP) (140). ZMP is an AMP analog that binds to the γ subunits of AMPK at the same sites as AMP and elicits the same effects of activation (141, 142). However, the potency of ZMP for AMPK activation is ∼20-fold lower compared with that of AMP (139). In addition, ZMP regulates several AMP-sensitive enzymes (143). Although useful as a biologic research tool, the combination of poor selectivity, low potency, and inadequate bioavailability (144) makes AICAR an unattractive clinical candidate. ZMP is an intermediate in purine metabolism, and, although ZMP rapidly accumulates intracellularly in most primary cell types, some immortalized cell lines have high rates of purine synthesis, and hence ZMP does not accumulate (138). This could offer an explanation as to why the effects of AICAR vary depending on cell type. In skeletal muscle, AICAR appears to induce comparable activation of α1- and α2-containing AMPK complexes and increases glucose uptake in an AMPK α2β2γ3 complex–dependent manner (125, 145, 146). Another AMP analog that has been shown to activate AMPK is 5-(5-hydroxyl-isoxazol-3-*yl*)-furan-2-phosphonic acid (C2) (147). C2 is a derivative of the prodrug compound-13 and activates AMPK with an EC_50_ in the low nanomolar range, which is ∼1000-fold lower than AMP (147). C2 is a partial agonist of α2-containing complexes and selectively protects α1 complexes against dephosphorylation (148). Despite being an AMP analog, C2 does not appear to modulate other AMP-sensitive enzymes, nor does it antagonize AMP on these enzymes (148). Interestingly, recent crystal structures reveal two C2 binding sites in the γ subunit distinct from the nucleotide sites (149), questioning whether C2 should be classified as an allosteric AMPK activator.

### Compounds that perturb the cellular AMP/ATP ratio

Because AMPK is activated by cellular increases in the AMP/ATP ratio, any mechanism that disrupts ATP generation will increase cellular AMP levels and ultimately activate AMPK. This complicates the search for attractive and specific AMPK activators because any compound that exerts an energetic stress on a cell is likely to activate AMPK. Hence, monitoring cellular stress and toxicity irrespective of selective AMPK activation is key when examining activators. The largest known classes of AMPK activators that work by this mechanism are inhibitors of mitochondrial ATP generation, such as metformin and berberine (mitochondrial respiratory chain complex I) (150, 151) and resveratrol (mitochondrial ATP synthase) (152, 153). Early studies showed that metformin acutely stimulates glucose uptake in skeletal myotubes (154). However, in mouse skeletal muscle, acute metformin treatment does not seem to alter glucose uptake despite modest activation of AMPK (155). Instead, chronic metformin treatment appears to enhance insulin-stimulated glucose uptake in an AMPK-dependent manner (155). Another compound in this class includes PT-1, which inhibits mitochondrial respiration independently of ATP synthase and appears to selectively activate γ1-containing complexes in skeletal muscle. Interestingly, this does not lead to increased glucose uptake in skeletal muscle (65). It will be useful to investigate how an indirect activator that acts by perturbing the cellular AMP/ATP ratio can activate only AMPKγ1-containing complexes. As mentioned previously, localization of the specific AMPK complexes may play a role in this selectivity. More than 100 less-characterized AMPK activators have emerged in the literature throughout the years (138). A substantial portion of these are natural plant products, and, although the mechanism of activation is unknown for the majority of these products (138), most naturally derived AMPK activators with known mechanisms, such as metformin, resveratrol, and berberine, are indirect activators. Thus, it can be speculated that a considerable fraction of these plant-derived activators belong to this subgroup of indirect AMPK activators.

### Allosteric activators

Of the large pool of AMPK activators in the literature, very few have been validated as direct allosteric activators with mechanisms of activation distinct from that of AMP. Of the compounds validated, even fewer have been tested in skeletal muscle. This section focuses on the compounds that have been explored in skeletal muscle. The first of such activators to be described was A-769662 (156). A-769662 binds in a pocket between the kinase domain on the α subunit and the carbohydrate-binding module on the β subunit (157) and does not alter the cellular AMP/ATP ratio (156). A-769662 activates AMPK both allosterically and by inhibiting dephosphorylation of AMPK on α-Thr172 (158). The seminal study on A-769662 showed that administration to ob/ob mice markedly lowered plasma glucose levels (156). Interestingly, although A-769662 activates β1-containing AMPK complexes in mouse skeletal muscle lysates, Treebak *et al.* (70) showed that A-769662–induced glucose uptake was dependent on mouse strain and appeared to occur through a wortmannin-sensitive mechanism. The majority of β1 in skeletal muscle lysates likely originate from nonmuscle cells, and hence the effect of β1 agonists in skeletal muscle tissue is questionable. Another recently identified allosteric activator, compound 991 (also referred to as ex229), binds to the same site as A-769662 but displays 5- to 10-fold higher potency (159). *In vitro* experiments have demonstrated that compound 991 binds 10 times stronger to AMPKβ1-containing complexes than to complexes containing β2 (159). Nevertheless, this compound activates both β1- and β2-containing complexes in mouse skeletal muscle and increases glucose uptake in myotubes by an AMPK-dependent mechanism (160). Two new AMPK activators described recently, MK-8722 and PF-739, demonstrate tremendous advances in developing selective AMPK activators with desirable potency and bioavailability (161, 162). Similar to 991 and A769662, these compounds display higher affinity toward β1, but have the ability to activate all AMPK complexes. These compounds robustly activate AMPK in skeletal muscle with EC50s in the nanomolar range. They increase skeletal muscle glucose uptake and lower blood glucose in rodents and nonhuman primates (161, 162). Reiterating the absence/low importance of β1 complexes in skeletal muscle, the β1-selective activator PF249 fails to activate AMPK in mouse skeletal muscle (161). The pharmacokinetic and pharmacodynamic relationships of these two novel compounds make them very attractive tools to study the effects of AMPK activation *in vivo*. Although the allosteric activators described in this review so far are synthetic compounds, another allosteric AMPK activator is salicylate, a natural plant product from willow bark and a derivative of acetyl salicylic acid (aspirin). Salicylate binds at the same site as A-769662 and predominantly activates β1-containing complexes (163). Interestingly, in rat skeletal muscle, salicylate activates α1- and α2-containing AMPK complexes to a comparable extent at 5 mM. This concentration of salicylate also induces a substantial increase in glucose uptake (164). It appears that most known allosteric activators of AMPK to date, despite showing affinity toward β1-containing complexes, can be regarded as pan activators in skeletal muscle.

The AMPK field has long sought after useful pharmacological tools to selectively activate AMPK. Although the traditional tools (*i.e.*, AMP analogs and compounds that alter the cellular AMP/ATP ratio) have provided vital insight into the biologic function of AMPK, their modes of activation make them unselective. Hence, the emergence of potent AMPK-selective small-molecule activators like compound 991, PF-739, and MK-8722 (159, 161, 162) hold high promise for the future. From a skeletal muscle–centric view, a new generation of isoform selective activators is highly warranted. Specifically, selective activation of γ3-containing complexes could have major implications not only for the understanding of skeletal muscle AMPK, but potentially for AMPK activation as a therapeutic strategy. So far, the strategy for small-molecule activation of AMPK has been to use a drug-binding pocket located between the α-subunit kinase domain and the β-subunit carbohydrate-binding module [also known as the allosteric drug and metabolite (ADaM) site]. Although this has proven effective for β-selective AMPK activation, this strategy is not viable for γ-selective activation. Currently, there are no crystal structures available of γ3-containing AMPK complexes, and hence we do not know whether there are suitable drug-binding pockets in these complexes. A potential strategy is to take advantage of one or more of the 4 AMP-binding sites on the γ subunit (165), an approach partly used by the AMPK activator compound-13. However, compound-13 directly competes with AMP (148) and as such directly competes with the biologic regulation of AMPK activity. Furthermore, it is uncertain whether such an approach permits γ3 selectivity over other γ isoforms. A recent publication on compound-13 revealed that this compound selectively activates γ1- and γ2-containing AMPK complexes (149), suggesting that there are indeed structural differences between the γ-subunits. Such differences may form the basis for γ3-selective AMPK activators in the future. Regardless, further understanding of the γ subunits in the complex are warranted to design the next generation of AMPK activators. As an alternative to customized small-molecule AMPK activators, it may be speculated that the drug-binding pockets of AMPK is a site for endogenous AMPK ligands, although these have not been identified. Therefore, it may be worth investigating the presence of physiologic hormones/proteins that activate AMPK *via* its drug-binding pockets. This may permit the generation of highly selective and viable direct AMPK agonists for therapeutic use.

## AMPK REGULATION BY MYOKINES

Exercise has proven to be effective for the prevention and treatment of metabolic disorders such as obesity, type 2 diabetes, and cardiovascular disease (166). This protective effect has been linked to the renewed interpretation of skeletal muscle as an endocrine organ (167). Several reports have shown that skeletal muscle produces and releases a variety of substances, called myokines, during exercise and contraction (168, 169). These myokines act as autocrine, paracrine, or endocrine factors and may mediate the beneficial effects on metabolic and physiologic responses to exercise in the skeletal muscle itself and in distant organs such as heart, lungs, brain, adipose tissue, and liver (167).

IL-6 is the most extensively studied myokine. Originally considered a proinflammatory cytokine because its plasma concentration is often elevated in patients with type 2 diabetes (170), it was later reintroduced as a therapeutic myokine because it was discovered that IL-6 is produced by contracting skeletal muscle and is released in high amounts into the circulation to exert its endocrine function (171–173). Additionally, IL-6 functions as an important autocrine factor in skeletal muscle through the maintenance of skeletal muscle glucose homeostasis during prolonged exercise and up-regulation of its own production (171–173). The relationship between AMPK and IL-6 has been extensively examined since MacDonald *et al.* (174) found a correlation between IL-6 release from muscle and tissue AMPKα2 activity after 60 min of exercise in humans. However, it remains controversial whether AMPK is involved in the secretion of IL-6 by skeletal muscle during exercise or, on the contrary, whether AMPK activity is modulated by the autocrine myokine action. Findings based on *in vitro* AICAR treatment have generated conflicting results. Some studies have documented a positive effect of AICAR on the myokine expression in C2C12 muscle cells (173) and depletion of IL-6 vesicles in skeletal muscle fibers (175), whereas other studies have shown a reduction in the IL-6 expression in myocytes (176) and its release by isolated skeletal muscle (177). Glund *et al.* (177) also demonstrated that the basal IL-6 release from oxidative muscle was elevated in AMPKα2 kinase dead and AMPKα1-KO mice models, indicating that IL-6 regulates its own release *via* AMPK in resting skeletal muscles. To complete the broad studies made using different transgenic mouse models of AMPK, it was recently demonstrated that AMPKα mdKO mice display elevated expression of IL-6 mRNA in gastrocnemius muscle, which might be due to mild skeletal muscle damage (72). On the other hand, IL-6 activates AMPK *in vitro* (178, 179) and *in vivo* (179, 180), as demonstrated in IL-6-KO mice, which exhibit decreased AMPK activity in skeletal muscle. Taken together, current observations do not provide a clear picture on the possible relationship between IL-6 and AMPK in skeletal muscle, and further research is needed to validate its manifestation. Although IL-6 is greatly implicated in mediating the health benefits of exercise and muscle regeneration after injury (181), higher systemic levels of the cytokine have been shown to induce muscle proteolysis (182). To the best of our knowledge, no involvement of AMPK in this detrimental effect has been suggested. Additionally, it has been recently proposed that the cytokine exhibits a dual pro- or anti-inflammatory effect depending on its downstream molecular pathway (183).

Other myokines produced and released by skeletal muscle are IL-8, IL-15, irisin, brain-derived neurotrophic factor (BDNF), and leukemia inhibitory factor (LIF) (184–188). These myokines have been less studied, and there are only a few studies linking them to AMPK. Lihn *et al.* (176) demonstrated that *in vitro* AICAR stimulation reduces IL-8 expression similar to IL-6 in human skeletal muscle cells. More attention has been given to the possible relationship between AMPK and IL-15. As such, IL-15 protein content has been found to be lower in skeletal muscle of AMPKα2 kinase dead mice in both control and high-fat diet (HFD) groups (189). In addition, AMPKβ1β2M-KO mice exhibit lower IL-15 levels in muscle and plasma (190). The same transgenic mouse model also displays reduced plasma irisin levels in the basal state (191). Moreover, it has been shown that irisin induces phosphorylation and activation of AMPK *in vitro* (192). Similarly, BDNF can act in an autocrine/paracrine manner to boost fat oxidation in skeletal muscle during contraction *via* AMPK (187). Finally, skeletal muscle LIF mRNA expression increases immediately after exercise and declines gradually during recovery (188). However, a definite link to AMPK has yet to be established. Interestingly, LIF has been shown to increase glucose uptake in skeletal muscle, although this does not seem to require AMPK (193).

Although it is well established that skeletal muscle expresses a variety of myokines, it is hard to believe that AMPK is the only mediator of their release. Considering the energy-preserving nature of AMPK, it may be speculated that skeletal muscle AMPK is involved in releasing myokines that facilitate restoration of myocellular energy homeostasis. If so, myokines such as IL-6, IL-15, BDNF, and LIF are possible candidates based on their promoting effects on glucose uptake and fatty acid oxidation in skeletal muscle as well as lipolysis in adipocytes (178, 187, 193–195).

## AMPK IN THE CONTROL OF MUSCLE GLUCOSE UPTAKE AND FATTY ACID OXIDATION

The first evidence linking AMPK to regulation of glucose uptake and fatty acid oxidation in skeletal muscle was provided in 1997 by Merrill *et al.* (9), who observed that AICAR not only increased AMPK activity in rat skeletal muscle but also enhanced muscle glucose uptake and fatty acid oxidation (9). Later observations in the AMPKα2 kinase dead mouse model provided genetic evidence to support a role of AMPK in regulating muscle glucose uptake but not fatty acid oxidation in response to AICAR stimulation (196, 197). The effect of AICAR on muscle glucose uptake was subsequently shown to be dependent on the α2β2γ3 complex (125, 145, 146). In contrast to AICAR, evidence linking AMPK to increased muscle glucose uptake and fatty acid oxidation during exercise and contraction remains inconclusive. Thus, the AMPK β1β2M-KO mouse model shows increased reliance on fat oxidation during treadmill exercise (74), whereas the AMPKα mdKO and LKB1-KO mouse models display decreased fat oxidation during treadmill exercise at the same relative intensity (73, 198). Moreover, exercise-induced muscle glucose uptake is impaired in AMPK β1β2M-KO mice but remains intact in AMPKα mdKO and LKB1-KO mice (73, 74, 198). Findings from several AMPK-deficient mouse models also suggest that *ex vivo* contraction-stimulated glucose uptake is intact (72, 125, 146, 199, 200) or only partially decreased (74, 145, 196, 201, 202) in skeletal muscle. Similarly, rates of fatty acid oxidation are not impaired in skeletal muscle from different AMPK-deficient mouse models during *ex vivo* contraction, although an overall reduction of fatty acid oxidation may be observed in skeletal muscle of AMPK-deficient mice compared with wild-type (WT) mice (73, 146, 197, 198). However, this reduction is associated with lower cluster of differentiation 36 (CD36) and/or fatty acid binding protein content in skeletal muscle of the AMPK-deficient mouse models (73, 198), which are thought to be important regulators of fatty acid uptake and oxidation (203). Although pharmacological activators of AMPK increase glucose uptake and fatty acid oxidation in skeletal muscle by an AMPK-dependent mechanism, AMPK is not or is only partially necessary for regulating these processes during muscle contractile activity. The reason for this discrepancy is not clear but may relate to redundant signaling during exercise or to the finding that AMPK regulates muscle glucose uptake and fatty acid oxidation during physiologic stimuli other than exercise that activates the complex in skeletal muscle (*e.g.*, food deprivation and hypoxia). Regarding the former, the AMPK-related kinase SNARK is activated in skeletal muscle in response to exercise, and knockout of SNARK is associated with impaired exercise-stimulated glucose uptake in skeletal muscle (81). Thus, it could be speculated that SNARK may compensate for the lack of skeletal muscle AMPK, thereby masking the effect of AMPK in regulating muscle glucose uptake during exercise. Measurements of SNARK activity in skeletal muscle depleted of AMPK activity could reveal insight into this notion.

To unravel a potential role of AMPK in regulating muscle glucose uptake and fatty acid oxidation, studies investigating downstream signaling of AMPK may provide clues. It has been suggested that AMPK enhances glucose uptake by stimulating GLUT4 translocation to the muscle surface membrane (196, 204). Although not fully described, AMPK-mediated glucose uptake may involve phosphorylation of downstream target TBC1D1, which has been proposed to regulate GLUT4 translocation (205). Hence, several studies using different AMPK-deficient models have shown that several phosphorylation sites on TBC1D1 are regulated by AMPK in skeletal muscle during *ex vivo* contraction (73, 74, 122, 206–208). Furthermore, phosphorylation of TBC1D1 increases in human skeletal muscle during exercise and has been found to correlate with AMPK heterotrimer–specific activity (71, 122, 207, 209). Taken together, these observations suggest a functional relationship between TBC1D1 and AMPK in regulating exercise-induced muscle glucose uptake by redistributing GLUT4 from the cell interior to the cell surface membrane. In support of this, glycolytic skeletal muscle from various TBC1D1-deficient mouse models exhibits impairments in exercise-, contraction-, and AICAR-stimulated glucose uptake (210–213). However, these mouse models display reduced levels of GLUT4 protein in skeletal muscle, and therefore they are not ideal for assessing the possible regulatory effects of AMPK on glucose uptake. In contrast, mouse tibialis anterior muscle overexpressing TBC1D1 mutated at 4 predicted AMPK phosphorylation sites (Ser231, Ser499, Ser660, and Ser700) expresses normal levels of GLUT4, although contraction-stimulated glucose uptake is reduced by ∼35% (208). Although reductions in glucose uptake after contraction may seem low in muscle overexpressing a mutated form of TBC1D1, only a portion of tibialis anterior muscle overexpresses TBC1D1 due to the transfection methodology used (*i.e.*, electroporation) (214). Thus, improvements in transfection efficacy could potentially induce a further reduction in glucose uptake during contraction, highlighting the likely importance of TBC1D1 in regulating AMPK-mediated glucose uptake. Interestingly, contraction- and exercise-induced glucose uptake is not compromised in skeletal muscle from TBC1D1 Ser231Ala KI mice (215, 216), suggesting that TBC1D1 needs to be phosphorylated at multiple sites by AMPK or modified otherwise to exert its effect on glucose uptake.

It is generally believed that AMPK regulates fatty acid oxidation in skeletal muscle through phosphorylation of acetyl-CoA carboxylase 2 (ACC2) on Ser212. This is supported by findings in ACC2 Ser212Ala KI mice in which AICAR fails to increase fatty acid oxidation in skeletal muscle (217). Phosphorylation of ACC2 Ser212 does not appear to play a role in regulating fatty acid oxidation during *ex vivo* contraction and exercise (218), providing evidence to support that AMPK-ACC2–independent pathways regulate fatty acid oxidation during muscle contractile activity. In addition, AMPK does not seem essential for increasing FAT/CD36 translocation and fatty acid uptake during *ex vivo* contraction (219), which may limit fatty acid oxidation during low to moderate contraction intensities (220). Considering these findings, the importance of AMPK in regulating muscle fatty acid oxidation during exercise appears sparse given the genetic evidence obtained from various transgenic mouse models. A role of AMPK in regulating muscle fatty acid oxidation in exercise recovery has been suggested based on reduced ACC activity and malonyl-CoA levels and enhanced fatty acid oxidation after a single bout of treadmill exercise (221). This is also in line with the notion that, although phosphorylation of AMPK returns to baseline quite quickly after exercise in skeletal muscle, downstream effects on substrates like ACC Ser212 are increased, which may enhance fatty acid oxidation early in the postexercise period (209, 222). This suggests that AMPK may regulate muscle fatty acid oxidation after exercise rather than during exercise.

## AMPK AND PROTEIN METABOLISM

**Figure 5.**
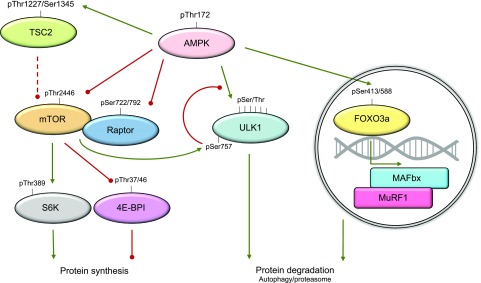
Role of AMPK in protein metabolism. Activation of AMPK increases phosphorylation of regulatory associated protein mTOR (Raptor) and mTORC1 itself as well as TSC2, an indirect inhibitor of mTORC1. This decreases the activity of S6 kinase (S6K) and inhibits the release of binding partner eukaryotic translation factor 4E (eIF4E) from eukaryotic translation initiation factor 4E-BP1, thereby diminishing protein synthesis. Besides its effect on protein synthesis, AMPK promotes autophagy and proteasomal-mediated protein degradation by phosphorylating ULK1 (Ser317/467/555/637/777 and Thr575) and FOXO3a, respectively. When phosphorylated, FOXO3a increases the expression of E3 ligases MAFbx and MuRF1. Green lines, promotive; red lines, inhibitory; dashed line, indirect. Adapted from Sanchez *et al.* (42).

Mammalian target of rapamycin complex 1 (mTORC1) is a well-known cell nutrient and energy sensor, as demonstrated by its ability to sense amino acids and regulate protein translation as well as autophagy, two major processes involved in cellular protein turnover (223, 224). mTORC1 regulates muscle mass development by controlling protein translation initiation *via* its two major downstream targets: the p70 ribosomal S6K and the eukaryotic initiation factor 4E binding protein 1 (4E-BP1) (225). mTORC1 activity is regulated by AMPK through phosphorylation of tuberous sclerosis complex 2 (TSC2) at Thr1227 or Ser1345, which improves the ability of TSC2 to inhibit mTOR activity (226). Alternatively, AMPK can directly phosphorylate mTOR Thr2446 to inactivate the complex, which prevents Akt-mediated phosphorylation of mTOR Ser2448, resulting in inhibition of translation initiation (227, 228). In addition, AMPK phosphorylates Raptor on Ser722 and Ser792, resulting in mTORC1 inhibition (229). Hence, increased energy demand dampens protein synthesis through a balance between the AMPK and mTOR signaling pathways (**Fig. 5**). Interestingly, rats treated with AICAR have reduced skeletal muscle protein synthesis, which is associated with down-regulation of the Akt-mTOR pathway and its downstream targets S6K and 4E-BP1 (11). Independently of mTORC1, AMPK may also inhibit translation elongation through direct phosphorylation of eEF2k (230).

Although AMPK activation inhibits mTORC1 to regulate protein synthesis, it has recently been shown that suppression of S6K activity promotes AMPK signaling (231). Indeed, the interplay between the AMPK and mTORC1 signaling pathway is tightly regulated by several upstream signals but also by several negative feedback loops operated by different kinases, such as S6K on insulin receptor substrate and AMPK (231–236). As such, AMPKα mdKO mice exhibit increased muscle mass with normal body weight compared with WT littermates, indicating a role of AMPK for controlling muscle growth (72). On the other hand, AMPK β1β2M-KO mice have normal skeletal/cardiac muscle weights as compared with their WT littermates (74), which may suggest that alternative or compensatory mechanisms might compensate for AMPK deficiency in AMPK β1β2M-KO mice. Although still to be investigated, AMPK activity could also be important for the maintenance of muscle protein turnover and growth in conditions of high energy demand such as exercise, caloric restriction, muscle regeneration, and repair (74).

AMPK is also known for regulating muscle protein degradation, which serves as a source of amino acids that can be used for energy production by various other organs. The ATP-dependent ubiquitin–proteasome system and the autophagy–lysosomal pathway are the major pathways for mediating protein degradation. The former involves a cascade of enzymatic reactions that tag substrate proteins with ubiquitin chains for degradation by the 26S proteasome. This system involves the activity of E3 ubiquitin ligases, which confer substrate specificity for ubiquitination and degradation. Two major E3 ligases have been described to be important for muscle atrophy: atrogin-1/muscle atrophy F-box (MAFbx) and muscle RING finger 1 (MuRF1) (237, 238). AMPK has been shown to phosphorylate forkhead box protein O3a (FOXO3a) at multiple sites, including Ser413 and Ser588 (239), which seems to control the transcription of the E3 ligases MAFbx and MuRF1, promoting muscle protein degradation (72, 240, 241) (Fig. 5). During starvation-induced atrophy, FOXO3a also regulates the transcription of several proteins involved in the autophagic machinery (242, 243).

Autophagy lysosomal–dependent protein degradation is an evolutionarily conserved recycling pathway for maintaining cell metabolism and protein turnover. There are three different types of autophagy. In macroautophagy, proteins, organelles, and cytosolic material are sequestered in an autophagosome to be degraded after the autophagosome fuses with lysosomes. In endosomal microautophagy, portions of cytoplasm are taken up into the lumen of the lysosome to be degraded. The third mechanism is chaperone-mediated autophagy, where substrate proteins are recognized by chaperone proteins, which facilitate lysosomal membrane crossing through a translocation complex (244). Macroautophagy is robustly activated in response to nutrient deprivation and inhibited by nutrient-sensor proteins such as mTORC1 (245–248). In the last decades, several studies have identified AMPK as a main regulator of autophagy in response to food deprivation, exercise, and stressors. AMPK directly phosphorylates multiple sites in uncoordinated 51-like kinase 1 (ULK1) (Ser317, Ser467, Ser555, Thr575, Ser637, and Ser777) and promotes ULK1 function in autophagy (249–252). These observations, in addition to studies in ULK1 knockout cells, demonstrate the functional importance of ULK1 phosphorylation by AMPK for induction of autophagy. mTORC1 also regulates ULK1 and autophagy by directly phosphorylating ULK1 on Ser757, which disrupt AMPK–ULK1 interaction. These results suggest that AMPK and mTORC1 have opposite roles on autophagy regulation depending on the cellular energy status (Fig. 5). However, recent studies in humans and mice suggest that AMPK alone may not be sufficient to activate autophagy in response to exercise and food deprivation in skeletal muscle (253, 254). Autophagy-specific gene proteins (Atgs) are essential mediators of autophagy based on their role in controlling the formation of the autophagosome. Activation of the autophagic enzyme machinery (*e.g.*, Atg5, Atg7, and nutrient-deprivation autophagy factor-1) is required for skeletal muscle remodeling and proper mitochondrial function (255–259); thus, skeletal muscle autophagy is an essential process required for maintaining muscle function and whole-body energy metabolism (260). Although the role of AMPK on chaperone-mediated autophagy has not been extensively studied, recent evidence from skeletal muscle suggests that AMPK may be implicated in the regulation of chaperone-mediated autophagy to degrade specific proteins. Indeed, AMPK phosphorylates perilipin 2 (PLIN2), which increases PLIN2 degradation (261). However, the physiologic role of AMPK regulating PLIN2 protein abundance and chaperone-mediated autophagy in skeletal muscle remains to be investigated in detail. During healthy conditions, skeletal muscle is required to supply gluconeogenic amino acids (such as alanine) for the maintenance of blood glucose levels during prolonged food deprivation (262). It was recently demonstrated in mice that the glucose–alanine cycle is regulated by AMPK-mediated autophagy in skeletal muscle, which increases alanine production for hepatic gluconeogenesis to avoid hypoglycemia during food deprivation (263). Taken together, these observations suggest that AMPK acts as a regulator of protein turnover in skeletal muscle.

Different stressors, such as starvation, exercise, and hypoxia, strongly perturb the function of mitochondria and lead to the production of large amounts of reactive oxygen species (ROS), which are harmful to proteins, lipids, and DNA and impair cellular ATP production. Thus, damaged mitochondria must be either repaired or removed. Recent evidence suggests that macroautophagy can be organelle specific in order to remove dysfunctional organelles such as mitochondria (mitophagy) (264). In atrophying muscle, the mitochondrial network is severely remodeled after food deprivation or denervation, and autophagy *via* Bnip3 contributes to mitochondrial remodeling (265). In 2011, Egan *et al.* (249) identified AMPK as a regulator of mitophagy connecting energy sensing to mitochondrial degradation *via* ULK1 phosphorylation. In support of this, AMPK-dependent phosphorylation of ULK1 was recently shown to be critical for translocation of ULK1 to mitochondria and for mitophagy in response to hypoxic stress (266). In addition, AMPK may prepare mitochondria to undergo mitophagy by stimulating mitochondrial fission *via* phosphorylation of mitochondrial fission factor and facilitating isolation of damaged mitochondria structure (267, 268). Thus, AMPK emerges as a master regulator of mitochondrial homeostasis, coupling mitochondrial dynamics to mitophagy. Interestingly, skeletal muscle of AMPKβ1β2M-KO mice has fewer but larger mitochondria compared with WT mice (74). Furthermore, it has recently been shown that acute exercise-induced mitophagy is regulated through AMPK-dependent phosphorylation of Ulk1 in skeletal muscle (269). This provides evidence to support that AMPK is indeed involved in removal of dysfunctional mitochondria to preserve skeletal muscle function.

It is well known that in skeletal muscle, exercise and starvation induce physiologic levels of ROS acting as signaling molecules (270–272). In humans, the use of antioxidants can prevent some of the beneficial effects of exercise training, such as enhanced insulin sensitivity (273). Although the role of ROS in these contexts needs further investigation, induction of fast autophagy by ROS may be a mechanism by which starvation and exercise provide nutrients but also remove dysfunctional mitochondria in skeletal muscle. On one hand, ROS and reactive nitrogen species activate ataxia telangiectasia mutated kinase to recruit LKB1/AMPK and/or TSC1/2 to inhibit mTORC1 and activate autophagy/mitophagy (274–277). On the other hand, AMPK may also directly sense cytosolic redox homeostasis because AMPK has been proposed to be activated upon H_2_O_2_ exposure, particularly through S-glutathionylation of oxidized cysteines within the AMPKα and AMPKβ subunits (278). Antioxidants that block basal mitochondrial ROS production, induced by acute nutrient starvation, also reduce AMPK phosphorylation and inhibit induction of autophagy in skeletal muscle (279). Starvation-induced AMPK activation and autophagy seem to be dependent on mitochondrial ROS based on findings in cell cultures showing that overexpression of SOD2 or stimulation with ROS scavenger N-acetyl-cysteine attenuates activation of AMPK and autophagy during starvation (280). In addition to mitophagy, recent studies have shown that accumulation of ubiquitinylated proteins activates AMPK to induce autophagy in mouse embryonic fibroblasts and macrophages, and similar processes could also be important for skeletal muscle homeostasis (281). Altogether, these studies indicate that AMPK acts as a redox sensor in order to activate autophagy and mitophagy to promote protein degradation and remove dysfunctional mitochondria, thus avoiding toxicity due to high production of ROS and reactive nitrogen species in mitochondria.

Taken together, these findings show that AMPK acts on anabolic and catabolic processes to regulate muscle protein metabolism. On one hand, it can decrease muscle mass by regulating protein synthesis; on the other hand, it can promote ubiquitin-proteasome and autophagy in response to nutrients and changes in the redox states (274–281).

## AMPK IN THE CONTROL OF MUSCLE MITOCHONDRIAL FUNCTION AND CELLULAR ENERGY CHARGE

AMPK relays the signal of low energy charge through phosphorylation of proteins important for the ability of the cell to adapt to changes in energy availability (282). Thus, AMPK signaling to target proteins constitutes a cellular memory of energy stress. The ability of a cell to match substrate utilization with demand involves a coordinated regulation of genes encoding mitochondrial proteins, a process known as mitochondrial biogenesis (283). In response to repeated metabolic stress, AMPK orchestrates a cellular response to ensure sufficient mitochondrial adaptation, and various AMPK transgenic and KO mouse models have consistently shown altered capacity to regulate mitochondrial biogenesis in skeletal muscle compared with WT littermates (15, 72, 74, 128, 284–286). In skeletal muscle, AMPK controls mitochondrial biogenesis through PGC-1α and nuclear respiratory factor 1 (15). AMPK has been shown to phosphorylate and activate PGC-1α (287), which drives expression from the PGC-1α promoter itself and from a multitude of metabolic and mitochondrial genes. PGC-1α activity is also controlled by lysine acetylation mediated by general control nonderepressible 5 (288) and deacetylation mediated by NAD-dependent sirtuin1 (SIRT 1) (289, 290).

**Figure 6.**
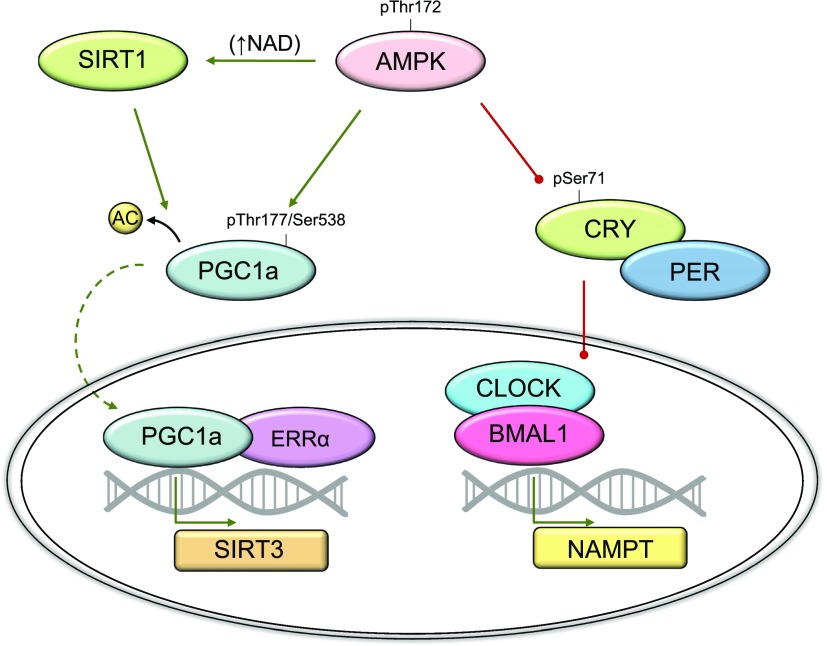
Role of AMPK in mitochondrial function and cellular energy charge. Activation of AMPK enhances phosphorylation of PGC-1α. In addition, AMPK facilitates activation of SIRT1 by increasing intracellular NAD concentrations, leading to deacetylation and activation of PGC-1α. After translocation to the nucleus, PGC-1α interacts with ERRα to increase activity of the SIRT3 promoter, causing an increase in mitochondrial biogenesis. AMPK has been proposed to regulate mammalian NAMPT gene expression *via* phosphorylation of CRYPTOCHROME (CRY), which together with PERIOD (PER) forms an inhibitory complex of CLOCK/BMAL1. When phosphorylated, CRY is degraded, allowing NAMPT transcription by CLOCK/BMAL1. Green lines, promotive; red lines, inhibitory.

Similarly to the AMP/ATP ratio, the ratio between the oxidized and reduced forms of NAD constitutes an index of cellular energy charge, and activation of AMPK with AICAR in C2C12 muscle cells has been found to increase SIRT1 activity by increasing NAD levels and thereby promote deacetylation of PGC-1α (291). Although the mechanism by which cytosolic/nuclear NAD levels increase in response to AMPK activation is not completely clear, muscle contraction has been shown to increase cytosolic and mitochondrial NAD levels as well as the NAD/NADH ratio (292). Moreover, disruption of AMPK signaling has profound effects on food deprivation– and exercise-induced SIRT1-dependent activation of PGC-1α signaling in skeletal muscle (293). This indicates a molecular circuit involving AMPK and NAD-dependent sirtuin signaling important for cellular metabolic function (**Fig. 6**).

Another level of control of PGC-1α expression is exerted through AMPK-mediated phosphorylation and SIRT1-mediated deacetylation of FOXO3 required for full transcriptional control of the PGC-1α gene (239, 291, 294). Although FOXO3 has been linked to initiation of autophagy in skeletal muscle (295), FOXO3 has also been shown to be important for muscle mitochondria to adapt to nutrient restriction (296). Collectively, regulation of mitochondrial biogenesis through PGC-1α in response to fluctuations in muscle energy levels appears to require several phosphorylation and deacetylation events.

The NAD/NADH ratio is thought to increase in mitochondria in response to metabolic stress (*e.g.*, muscle contractions) when oxidation of NADH is not matched by substrate influx resulting in NAD generation (292, 297). As a consequence, SIRT3 is activated, resulting in a priming of mitochondria to increase ATP production. Overexpression of SIRT3 in mouse skeletal muscle has been shown to induce an oxidative fiber type switch (type II to type I), to enhance energy expenditure, and to decrease muscle mass (298). Although some of the observed phenotypes associated with SIRT3 overexpression may be beneficial for metabolic health, these observations suggest that expression levels of SIRT3 should ideally be coordinated with other mitochondrial proteins. Indeed, in mouse muscle myotubes and brown adipocytes, PGC-1α has been shown to control SIRT3 expression. PGC-1α increases the activity of the SIRT3 promoter through an interaction with estrogen-related receptor (ERR)α and subsequent binding to an ERR binding element (299, 300) (Fig. 6). Moreover, mitochondrial biogenesis induced by overexpressing PGC-1α in C2C12 myotubes has been found to be mediated through SIRT3 (299). Consistent with the idea that PGC-1α regulates SIRT3 gene expression in skeletal muscle, SIRT3 mRNA and protein levels were found to be reduced by 50% in resting mouse muscle from PGC-1α knockout mice (286). Furthermore, there are clear indications that the described signaling axis between AMPK and PGC-1α includes SIRT3. Thus, overexpression of AMPK in primary mouse hepatocytes results in elevated levels of SIRT3 mRNA (301), and the exercise training–induced increase in SIRT3 protein abundance in mouse skeletal muscle is completely dependent on AMPK (286).

Knockout of the upstream AMPK kinase LKB1 in skeletal muscle affects the abundance of mitochondrial enzymes (99). In cultured liver cells, SIRT1 has been shown to deacetylate LKB1, resulting in its translocation to the cytosol where it activates AMPK (302). Moreover, the ability of AICAR to activate AMPK was blunted in primary myoblasts and hepatocytes from SIRT1 knockout mice (303). In contrast to these observations, AMPK activation measured by phosphorylation is not affected in response to an acute bout of exercise in skeletal muscle–specific SIRT1-KO mice (304). It is possible that secondary adaptations to muscle-specific deletion of SIRT1 could mask the true effects of SIRT1 to potentiate AMPK activation, but further studies are required to verify this potential feedback mechanism in skeletal muscle. In various human cultured cell lines, binding sites for FOXO3 have been found in the promoter region of LKB1, and AMPK activation induced by energy stress has been shown to increase LKB1 mRNA levels (305, 306). Whether a similar feedback mechanism is present in skeletal muscle has not been investigated.

Sirtuins break down NAD to nicotinamide (NAM) and 2-*O*-acetyl-ADPR. NAM negatively feeds back to inhibit these enzymes (307) and must be converted back to NAD to maintain sirtuin enzymatic activity. The rate-limiting enzyme in the main NAD salvage pathway in mammalian cells is nicotinamide phosphoribosyltransferase (NAMPT), accounting for most of the NAD generated in the cell, (308, 309). NAMPT converts NAM to nicotinamide mononucleotide, which is converted to NAD by nicotinamide mononucleotide adenylyltransferase, of which 3 isoforms exist (310). Thus, NAMPT may be important for the ability of cells to transmit signals associated with limited nutrient availability. In skeletal muscle, AMPK is important for maintaining NAMPT protein levels, and the AICAR-induced increase in NAMPT protein abundance is completely dependent on the AMPKα2 subunit (311). Furthermore, circadian oscillation in NAMPT mRNA expression is markedly blunted in skeletal muscle from whole-body AMPKα1- and AMPKα2-KO mice (312), and expression of core clock components is dysregulated in response to AICAR in skeletal muscle from AMPKγ3-KO mice (313). These findings indicate that circadian regulation of metabolism integrates nucleotide sensing and regulation of NAD salvage capacity in skeletal muscle and implies that AMPK may directly regulate NAMPT expression in this tissue. Mammalian NAMPT gene expression has been found to be under the control of the clock machinery (CLOCK/BMAL1) (314, 315), and AMPK has been shown to directly phosphorylate CRYPTOCHROME at Ser71, which, together with PERIOD, forms an inhibitory complex of CLOCK/BMAL1 (316). Phosphorylation of CRYPTOCHROME by AMPK results in its ubiquitination and degradation, allowing clock-controlled genes (*e.g.*, NAMPT) to be transcribed (Fig. 6) (316). It is assumed that the regulation of the circadian clock in muscle cells is regulated as described above. However, this needs further investigation.

AMPK appears to have profound effects on the ability of cells to adapt to conditions of low energy availability. Signaling through PGC-1α, to mediate changes that enable proper mitochondrial adaptation, requires fine-tuned feedback loops involving consumption of NAD as a substrate in SIRT1- and SIRT3-dependent reactions. Activation of AMPK not only increases NAD levels but also maintains sufficient NAD regeneration capacity by controlling abundance of NAMPT. Although NAD regeneration capacity in young, healthy skeletal muscle may not be limiting for mitochondrial function and muscle health (317), levels of NAD in skeletal muscle decline with age (318, 319) and are suppressed in mitochondrial myopathies (320). Thus, targeting AMPK or NAD salvage systems may prove useful for counteracting age-related reductions in muscle function.

## AMPK IN EXERCISE RECOVERY

After exercise, AMPK activity and downstream signaling decrease to levels observed in resting muscle typically within 3–7 h (131, 209, 222, 321, 322). The exact mechanism regulating AMPK activity in skeletal muscle several hours into recovery from a single bout of exercise is incompletely understood. However, it does not seem to involve changes in skeletal muscle adenosine nucleotide concentrations because these have been shown to return to pre-exercise levels 10 min after exhaustive exercise (323). Lower glycogen levels may account for some of the elevated AMPK activity observed in skeletal muscle after exercise but probably not all because AMPK activity reverses to resting levels before muscle glycogen levels have been fully resynthesized (131). Other factors that might explain elevated AMPK activity in exercise recovery include changes in heterotrimer localization and/or conformation that render the complex less sensitive for upstream phosphatases. In addition, activation of AMPK in nonmuscle cells situated or recruited to the muscle tissue could explain the slow reversal of AMPK activity observed in skeletal muscle after exercise.

### AMPK-dependent regulation of muscle gene expression after exercise

In recovery from a single bout of exercise, the transcriptional activity of genes coding for key metabolic proteins increases, which is followed by an increase in corresponding mRNA (324). The repeated activation of these transient stimuli by regular exercise may result in the beneficial adaptations of exercise training (325). The effect of exercise-induced AMPK activation on the gene expression response appears to involve a number of cellular transcription factors and coactivators, including PGC-1α (326). AMPK activation has been shown to increase gene expression of the 2 key glucose-handling intermediates, GLUT4 and HKII, by regulating histone deacetylase 5 (HDAC5) and cAMP response binding-element protein, respectively (327, 328). As such, a single injection of AICAR that elevates AMPK activity in skeletal muscle has been shown to increase gene expression of PGC-1α, HKII, and GLUT4 in WT mice but not in skeletal muscle of AMPKα2-deficient mice (329, 330).

Acute exercise induces a similar gene response at the level of GLUT4, PGC-1α, and HKII in WT and AMPKα2-deficient mice, suggesting that exercise-induced gene expression is orchestrated independently of AMPK (329, 330). These findings indicate that repetitive AMPK activation by pharmacological means is sufficient to increase gene expression, whereas signaling parameters other than AMPK may be essential for exercise-induced gene expression. An alternative scenario could be that redundant signaling, through the remaining AMPKα1 complexes in the transgenic model used, may be sufficient to induce these metabolic changes. Recently, exercise- and training-induced gene expression was investigated in AMPKα mdKO mice (331). In this study, acute exercise–induced gene response at the level of COX-I, GLUT4, and VEGF increased in an AMPK-dependent manner. On the other hand, the acute exercise–induced gene response for HKII, CD36, and FATP1 increased similarly in skeletal muscle from AMPKα mdKO and WT littermates. Collectively, these observations suggest that AMPK is essential for the acute exercise-induced up-regulation of some but not all genes encoding key metabolic proteins. However, although AMPK seems to be essential for up-regulation of gene expression for some key metabolic proteins in response to acute exercise, this seems not to be the case for training-induced adaptations.

### AMPK and postexercise muscle insulin sensitivity

Skeletal muscle glucose uptake increases markedly during exercise in an intensity-dependent manner and reverses to resting levels within a few hours of recovery (332). However, the ability of insulin to stimulate muscle glucose uptake is markedly enhanced in the period after exercise (333–336). The increase in insulin sensitivity of prior exercised skeletal muscle can persist for up to 48 h, facilitating muscle glycogen replenishment in recovery from exercise (336, 337). Insulin and exercise increase muscle glucose uptake by increasing GLUT4 translocation to the plasma membrane (338–340), and it has therefore been proposed that prior exercise increases muscle insulin sensitivity by potentiating GLUT4 translocation in response to insulin (341). The underlying mechanism responsible for enhancing postexercise insulin sensitivity seems to be induced locally within the prior exercised muscle (342) and appears to be independent of elevated activity of the proximal insulin signaling cascade (343–347). Insulin and exercise signaling converge at the level of TBC1D4 (348–351), a Rab-GTPase involved in the regulation of GLUT4 translocation. It has been proposed that phosphorylation of TBC1D4 may provide a mechanism for exercise-induced improvements in muscle insulin sensitivity. Thus, after insulin stimulation, phosphorylation of TBC1D4 increases to higher levels in prior exercised muscle compared with rested muscle, concomitant with enhanced muscle insulin sensitivity (348, 352). A direct link between AMPK and TBC1D4 becomes evident by observations demonstrating that pharmacological activation of AMPK by AICAR in incubated muscle leads to increased phosphorylation of TBC1D4 (351), an effect that is blunted in AMPK-deficient mice (353). Furthermore, it has been demonstrated that recombinant AMPK increases TBC1D4 phosphorylation in cell-free assays (353) and that phosphorylation of Ser704 on TBC1D4 increases in response to AICAR and contraction in an AMPKα2-dependent manner.

**Figure 7.**
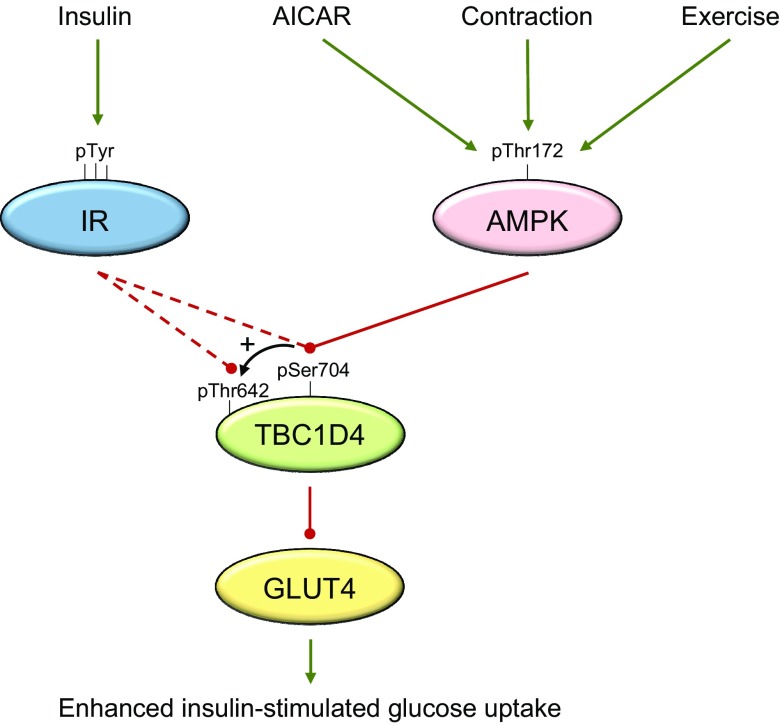
Involvement of AMPK in regulating improvements in muscle insulin sensitivity after exercise. AMPK phosphorylates TBC1D4 at Ser704 in response to AICAR, contraction, and exercise. This likely primes TBC1D4 for insulin signaling at Ser704 and Thr642, which enhances translocation of GLUT4 to the cell surface membrane, leading to improvements in insulin-stimulated muscle glucose uptake. Green lines, promotive; red lines, inhibitory; black line, priming; dashed lines, indirect.

Supporting the role of AMPK in regulating muscle insulin sensitivity, prior pharmacological AMPK activation by AICAR enhances insulin sensitivity in rat skeletal muscle (354). This effect is absent in AMPKα mdKO mice but is seen in concert with increased site-specific phosphorylation of TBC1D4 in WT mice (129). The latter study furthermore demonstrated that prior AMPK activation by AICAR increased AMPK activity of the α2β2γ3 complex and that the insulin-sensitizing effect of prior AICAR stimulation was dependent on the AMPKγ3 subunit (129). In parallel, it was recently demonstrated that prior *in situ* contraction and treadmill exercise increase muscle and whole-body insulin sensitivity in WT mice but not in AMPKα mdKO mice (355). Furthermore, these findings were associated with enhanced insulin-stimulated phosphorylation of TBC1D4 at Thr642 and Ser704 in WT mice. Taken together, these findings suggest that AMPK activation is necessary to enhance muscle insulin sensitivity after acute exercise and muscle contraction (**Fig. 7**). However, exercise activates a broad range of signaling molecules that may play an important role in improving muscle insulin sensitivity after exercise. Yang and Holman (356) reported that insulin stimulation increases GLUT4 exocytosis, whereas AMPK activation induced by oxidative stress inhibits GLUT4 endocytosis in cardiomyocytes. In human skeletal muscle, heterotrimer-specific activation of AMPK in recovery from exercise has recently been demonstrated (209), implying that enhanced AMPK activation in recovery from exercise may improve insulin sensitivity by delaying GLUT4 endocytosis. More research is needed to address the heterotrimeric regulation of AMPK in recovery from exercise and to transform these observations to physiologic endpoints, such as insulin sensitivity in human skeletal muscle.

### AMPK in the regulation of the intracellular fate of glucose in skeletal muscle

The breakdown of muscle glycogen during exercise increases in an intensity-dependent manner (357). Upon recovery from exercise, glycogen synthesis is of high metabolic priority in expense of increased lipid oxidation (358, 359). Glycogen synthase (GS), the rate-limiting enzyme in glycogen synthesis, catalyzes the incorporation of UDP glucose into branching glycogen polymers (360). The activity of GS is regulated by the allosteric activator glucose-6-phosphate and covalently by phosphorylation at multiple inhibiting residues (361, 362). AMPK has been identified as a site 2 (Ser7) GS kinase *in vitro* (363), and in rodent skeletal muscle phosphorylation of GS increases in response to AMPK activation by AICAR in an AMPKα2-dependent manner (10). This event decreases GS activity, however, because AICAR increases glucose uptake the subsequent intracellular rise of glucose-6-phosphate has been shown to overrule GS inhibition *via* covalent AMPK-induced phosphorylation (364).

The AMPK β subunit contains a GBD that allows the kinase to bind to glycogen (56, 106), suggesting that AMPK, in addition to its energy sensing role, acts as a glycogen sensor (365). According to this model, a pool of AMPK is responsive to changes in muscle glycogen content. Thus, during high glycogen levels, activity of this AMPK pool increases, consequently decreasing GS activity by phosphorylation of site two. Conversely, when glycogen levels are low, activity of this AMPK pool decreases, which increases GS activity due to decreased phosphorylation of site two (365). Although this scenario is based on a subfraction of AMPK, it contradicts the general observations of glycogen as a negative regulator of AMPK activity (106, 107, 109). Furthermore, recent observations indicate that carbohydrate binding to the GBD reduces AMPK activation by increasing accessibility for α-Thr172 dephosphorylation (366). The subcellular localization and regulation of different AMPK complexes according to muscle glycogen content may explain some of these discrepancies. More recently, a novel phosphorylation site has been identified (β-Thr148) in the GBD (111). Thus, in HEK293T cells autophosphorylation of β-Thr148 prevents AMPK binding to glycogen. Low glycogen levels induced by glucose deprivation or AMPK activation by AICAR increase phosphorylation of β-Thr148, suggesting that the binding affinity of AMPK to glycogen is regulated in response to glycogen content and AMPK activation. The physiologic relevance of these findings needs to be tested in mature skeletal muscle where glycogen serves as an essential source of energy.

Mutation of the AMPKγ3 subunit, leading to chronic activation, promotes excessive glycogen storage in mouse (125), pig (124), and human skeletal muscle (123). In line with this, genetic deletion of AMPKγ3 or overexpression of an AMPKα2 kinase dead construct in skeletal muscle markedly reduces glycogen resynthesis after exercise (125, 367). Based on these observations, it would be interesting to investigate whether a reduction in glycogen resynthesis is caused by the lack of an AMPK-dependent increase in muscle insulin sensitivity for glucose uptake after exercise.

Exercise has a major impact on substrate utilization in the postexercise period. Thus, fat oxidation is enhanced, whereas glucose oxidation is suppressed in favor of muscle glycogen resynthesis (359). Several lines of evidence suggest that AMPK is involved in the regulation of glucose oxidation in skeletal muscle. As such, AMPK activation by AICAR in rat skeletal muscle has been shown to enhance glucose oxidation in concert with elevated activity of pyruvate dehydrogenase (PDH), a protein regulating the entry of glucose into the tricarboxylic acid cycle for oxidation (368). In contrast, PDH activity is increased in glycolytic muscle from AMPKα2-KO mice compared with WT littermates, albeit muscle PDH protein abundance is reduced by ∼20% in AMPKα2-KO mice (369). In line with this observation, glucose oxidation in recovery from *in vitro* muscle contraction is increased in AMPKγ3-KO mice compared with WT littermates, indicating that the presence of AMPK is required for suppression of glucose oxidation, which may become highly relevant in exercise recovery (369). Pyruvate dehydrogenase kinase 4 inhibits PDH activity by phosphorylation, and a study in primary cardiomyocytes has shown that pharmacological activation of AMPK by AICAR increases pyruvate dehydrogenase kinase 4 mRNA expression. Collectively, these observations indicate that AMPK is involved in the regulation of glucose oxidation in skeletal muscle, but whether AMPK-dependent regulation of glucose oxidation is important for fuel selection during exercise recovery has received surprisingly little attention. Supporting a role of AMPK in substrate selection, Fritzen *et al.* (370) observed enhanced glucose oxidation at a whole-body level in recovery from exercise in whole-body AMPKα2-KO mice. However, enhanced glucose oxidation early in recovery from exercise was not observed in mice with a muscle-specific deletion of AMPKα1/α2 (AMPKα mdKO) (73). Collectively, studies in a few AMPK-deficient mouse models indicate that skeletal muscle AMPK is involved in regulating the cellular fate of glucose in recovery from exercise at the level of insulin sensitivity (AMPK mdKO), glycogen synthesis (AMPK mdKO, AMPK kinase dead, and AMPKγ3 KO), and glucose utilization (AMPKα2 KO). Future studies should therefore aim to validate these findings in other AMPK-deficient models and integrate these aspects of AMPK regulation in recovery from exercise, ultimately transferring these observations to human settings.

## AMPK IN THE REGULATION OF TRAINING-INDUCED ADAPTATIONS

Preceding the first studies linking AMPK to the regulation of exercise training–induced adaptations, it was shown that prolonged feeding of rats with β-guanidinopropionic acid (β-GPA; creatine analog) increased mitochondrial biogenesis as well as GLUT4 and HKII expression (371, 372). Bergeron *et al.* (13) extended these findings by showing that AMPK was activated in rats fed β-GPA, and the following year mitochondrial and metabolic adaptations to chronic β-GPA injections were shown to depend on AMPKα2 because adaptations were abrogated in mice overexpressing a kinase-dead AMPKα2 construct (15). Using AICAR, the role of AMPK in the adaptive response was strengthened. Only WT and not AMPKα2-KO mice showed increased content of metabolic and mitochondrial proteins after 4 wk of subcutaneous AICAR injections (284). Collectively, these observations suggest that repeated artificial AMPK activation mimics some of the beneficial effects induced by regular exercise training (31). Similar to observations in the AMPKα-deficient mouse model, mice lacking PGC-1α do not increase skeletal muscle content of metabolic and mitochondrial proteins in response to chronic AICAR injections (373), indicating that AMPK may act through PGC-1α to induce exercise-like adaptations in skeletal muscle.

It is well established that endurance exercise training results in a more oxidative muscle phenotype, expressing increased content of metabolic and mitochondrial proteins (374, 375). Because AMPK is activated during exercise, it has been proposed that AMPK mediates the positive effects of exercise training on the skeletal muscle phenotype. However, solid evidence confirming a role of AMPK for *in vivo* exercise training–induced mitochondrial and metabolic adaptations is lacking. Indeed, in most studies (284, 285, 311, 331, 376, 377) but not all (286), exercise training–induced adaptations are not abrogated in mutant mice that lack functional AMPKα2 or both AMPKα1 and AMPKα2 in skeletal muscle. This was also demonstrated in a study comparing subgroups of AMPKα mdKO mice and WT littermates that had performed an equal exercise training volume during a 4-wk intervention period. Muscle content analyses revealed that only 1 out of 13 proteins measured increased more in WT than in AMPKα mdKO mice with exercise training (331).

A number of alternative mechanisms proposed to mediate exercise training-induced adaptations include the Ca^2+^/calmodulin-dependent protein kinase (CaMK) (378), p38 MAPK (379), and ROS (380), among others (381). These mechanisms could compensate for the loss of AMPKα activity in the AMPKα mdKO mouse model with respect to skeletal muscle adaptations induced by exercise and are discussed in the following sections.

The expression of GLUT4 is in part regulated by binding of the transcription factor myocyte enhancer factor 2 (MEF2) to the GLUT4 promoter (382, 383). In an unstimulated state, the activity of both MEF2 and the GLUT4 promoter is suppressed by binding of HDAC5 (327, 384, 385). However, phosphorylation of HDAC5 on Ser259 and Ser498 promotes the binding of 14-3-3 protein and the subsequent nuclear export of the complex (385, 386). Both of the HDAC5 serine residues are targeted by AMPK (327), and exercise enhances the binding affinity of MEF2 to the GLUT4 promoter (387). This delineates a pathway by which exercise stimulates the expression of GLUT4 through AMPK.

Nevertheless, acute exercise and exercise training have evoked similar adaptations in skeletal muscle GLUT4 gene expression and protein content, respectively, in WT and AMPKα2-deficient mice, in addition to other metabolic and mitochondrial markers (284, 329, 330). This prompted McGee *et al.* (388) to investigate whether HDAC5 was involved in a compensatory mechanism to maintain the adaptive response to exercise in skeletal muscle from AMPKα-deficient mice (29). They found that exercise-induced HDAC5 phosphorylation of Ser259 and Ser498 was maintained in AMPKα2 kinase dead mice. Another HDAC5 kinase, protein kinase D (PKD), displayed elevated phosphorylation of Ser498 in skeletal muscle from AMPKα2 kinase dead mice compared with WT littermates immediately after exercise. Using myoblasts stably expressing constitutive active or inactive forms of PKD, they further showed that this was sufficient to regulate the expression of a range of metabolic and mitochondrial genes. It is therefore possible that exercise training adaptations in AMPKα2-KO and AMPKα mdKO mice may be elicited by compensatory PKD regulation of HDAC5 phosphorylation. To verify this *in vivo*, a concomitant deletion of AMPKα2 and PKD would be necessary. Since the results of McGee *et al*. (331) were published, it has been shown that the acute exercise-induced increase in gene expression (including GLUT4) was ablated in skeletal muscle from AMPKα mdKO mice, albeit exercise training–induced adaptations were similar to that of WT mice. In keeping with the intact adaptive responses in AMPKα2-deficient mice (284, 329, 330), results from the AMPKα mdKO model indicate that compensatory elevated AMPKα1 levels in skeletal muscle of AMPKα2-deficient mice may compensate for the lack of AMPKα2 in acute exercise–induced but not exercise training–induced adaptations. Therefore, it may be necessary to delete AMPKα1, AMPKα2, and PKD concomitantly in mouse skeletal muscle to prevent exercise training adaptations of metabolic and mitochondrial proteins.

Another mechanism that may induce compensatory adaptations in AMPKα-deficient mice with exercise and exercise training includes the nuclear hormone receptor 4A (NR4A) subgroup. This subgroup is comprised of 3 orphan nuclear receptors: Nur77 (neuron-derived clone 77; NR4A1), Nurr1 (nuclear receptor related 1; NR4A2), and Nor-1 (neuron-derived orphan receptor 1; NR4A3), and findings have shown that at least 2 of the 3 receptors exert metabolic control in many tissues, including skeletal muscle (389). Interestingly, exercise potently stimulates the gene expression of the NR4A subgroup in human skeletal muscle (325, 390). The rise in catecholamine concentration during exercise (391) may confer at least some of the mechanisms behind the induction of the NR4A genes because *in vivo* β-adrenergic stimulation increases their expression (392–394). Nuclear receptor signaling provides an alternative to the AMPK–PGC-1α pathway in regulating the expression of genes related to metabolic control. Findings suggest that this alternative β-adrenergic–NR4A signaling pathway may be up-regulated in skeletal muscle from AMPKα mdKO mice during exercise. Thus, in response to an acute bout of treadmill exercise at the same relative intensity, blood epinephrine concentration increased 131% in AMPKα mdKO mice, compared with a 27% increase in the WT littermates (interaction *P* = 0.07) (73). Therefore, it seems likely that higher activation of β-adrenergic signaling in AMPKα mdKO mice compared with WT mice could induce compensatory mitochondrial and metabolic adaptations with exercise training in skeletal muscles of the AMPKα mdKO mice. Furthermore, in the nonexercised state, a microarray analysis of extensor digitorum longus muscle from AMPKα mdKO and WT mice shows that Nur77 signaling is up-regulated in the mdKO mouse model (73), perhaps in an attempt to maintain the basal expression of metabolic and mitochondrial proteins.

Currently, catecholamine concentrations have not been reported in studies of AMPK-deficient mice other than the aforementioned study. More studies are needed to determine the exact role of β-adrenergic signaling and NR4A nuclear receptors in adaptations to exercise training.

## AMPK AND EXERCISE PERFORMANCE

Given that AMPK in skeletal muscle is implicated in many of the pathways otherwise involved in muscle contraction and muscle adaptation to exercise, the fact that muscle AMPK is physiologically activated by exercise is not surprising. One question that has been extensively studied over the years is how important AMPK is for muscle force development and exercise performance. Most mouse models with muscle deletions for various subunits of AMPK have therefore been studied in the setting of exercise. All models generated with skeletal muscle loss of function or KO of the AMPKα2 catalytic subunit or its preferred regulatory partner β2 or the upstream AMPK kinase (LKB1) display a phenotype of decreased *in vivo* exercise performance (time to exhaustion and maximal running speed) when forced to run on a treadmill, whereas performance (accumulative distance) during voluntary exercise (wheel running) has been reported to be similar or reduced compared with WT littermates (**Table 2**). Often muscle from these models shows decreased glycogen content but also indices of lower oxidative capacity, mitochondria content/function, and fatty acid oxidation during exercise or contraction (72–74, 198, 331, 395, 396). These findings suggest that AMPK is important during development for obtaining a normal metabolic capacity and thus likely also a normal exercise capacity, at least in rodents.

**TABLE 2.** Exercise capacity in various transgenic AMPK mouse models

Mouse model name	Target protein	Type of manipulation	Tissue specificity	Background strain	Running capacity*^a^*		Reference
					Forced	Voluntary	
α1KO	AMPKα1	Loss of function	Whole body	C57Bl/6J-Sv129	=	Unknown	370
α1 DN, also results in knockdown of AMPKα2	AMPKα1	Loss of function	Skeletal muscle (HSA)	Unknown	↓	Unknown	488, 489
α2KO	AMPKα2	Loss of function	Whole body	C57Bl/6J-Sv129	↓	=	198, 284, 370, 490
α2 kinase dead	AMPKα2	Loss of function	Heart + skeletal muscle (MCK)	C57Bl/6J	↓	↓; =	196, 311, 376, 377, 395–397, 491, 492
α2iTG (inactivating mutation in α2)	AMPKα2	Loss of function	Heart + skeletal muscle (MCK)	FVB	↓	=	285, 493
LKB1 DN	LKB1	Loss of function	Skeletal muscle (HSA)	Unknown	↓	Unknown	489
skmLKB1-KO	LKB1	Loss of function	Skeletal muscle (Myf6)	Unknown	↓	↓	398
LKB1-KO	LKB1	Loss of function	Heart + skeletal muscle (MCK)	FVB	↓	↓	99, 198
β1M-KO	AMPKβ1	Loss of function	Heart + skeletal muscle (MCK)	C57Bl/6J	=	Unknown	74
β2M-KO	AMPKβ2	Loss of function	Heart + skeletal muscle (MCK)	C57Bl/6J	↓	Unknown	74
β1β2M-KO	AMPKβ1 + AMPKβ2	Loss of function	Heart + skeletal muscle (MCK)	C57Bl/6J	↓	↓	74
α mdKO	AMPKα1 + AMPKα2	Loss of function	Skeletal muscle (HSA)	C57Bl/6J-Sv129	↓	↓	72, 73, 331
HA-R70Qγ1 transgenic	AMPKγ1	Gain of function	Skeletal muscle (HSA)	FVB	↑	Unknown	126

DN, dominant negative; HSA, human skeletal actin; MCK, muscle creatine kinase; Myf6, myogenic factor 6. *^a^*The symbols ↓, and ↑ denote whether the respective genetic mutation does not affect, decreases, or increases the running exercise capacity compared with WT littermates, respectively.

Although most of these models exhibit decreased exercise capacity, the intracellular phenotype induced by lack of AMPK function varies considerably between different models. Although this may reflect potential compensatory effects in different mouse strains (72, 74), it may also hint at hitherto unknown functions of AMPK. Although induction of mRNA expression of key metabolic genes by a single exercise bout is compromised in AMPKα-deficient muscle, this does not translate to impaired adaptations at the protein level in response to exercise training (331). Thus, AMPK signaling seems dispensable for normal adaptation to exercise training for a range of metabolic parameters (284, 311, 331, 376, 377, 397, 398) as well as exercise training–induced improvements in running performance (331, 398). The interpretation of many of these observations with exercise training is impeded by the overall decreased exercise capacity in the transgenic models [as illustrated by Fentz *et al.* (331)], and thus no solid genetic evidence has been put forward supporting that AMPK in the adult muscle is a true mediator of adaptations to endurance exercise training.

Nonetheless, a growing and illegal market for substances activating AMPK and the discovery of AICAR in trash bins around hotels of the cycling teams at the 2009 Tour de France led the World Anti-Doping Agency (WADA) to add any substances activating AMPK to the prohibited list (399). Besides these behavioral observations, the arguments from WADA were exclusively based on studies performed on animal models indicating ergogenic effects of AICAR treatment (400). In clinical trials, AICAR has been tested for minimizing damage after cardiac reperfusion (401), and the metabolic consequences of AICAR infusion have been studied in a few human experiments, apparently without major effects on AMPK activity in skeletal muscle (144, 402, 403). Therefore, the action of WADA is based on weak scientific evidence from animal models and virtually none from human experimentation. However, because the pharmaceutical industry continues to search for novel specific AMPK agonists combating diseases like diabetes and cancer, the availability of such substances is likely going to increase, and the (mis)use of such in the hope for ergogenic purposes will likely follow.

From the abovementioned possible roles of AMPK in muscle function and metabolism, one might predict that AMPK activation promotes exercise endurance. It might also promote fuel restoration in recovery from exercise, and it may be important in recovery from skeletal muscle injury. On the other hand, AMPK activation might impose a challenge for myofibrillar protein synthesis and hypertrophic responses to certain exercise training programs.

In the late 1960s, exercise physiologists began to illuminate the importance of pre-exercise muscle glycogen content and the importance of glucose/glycogen sparing (elevated fatty acid oxidation) during exercise as primary factors determining performance in endurance type of exercises. Thus, a muscle phenotype with high vascularization, high mitochondria capacity, potentiated/optimal oxidative handling of fatty acids, elevated glycogen content, and perhaps a fiber type distribution toward MyHC I and IIA were predicted to benefit exercise endurance. The question is then whether AMPK activation promotes such muscle phenotype.

Family members carrying a PRKAG3-activating mutation (AMPKγ3-R225W) provide the only human evidence so far that chronic AMPK activation leads to elevated skeletal muscle glycogen levels, decreased intramuscular triglyceride, and indices of increased oxidative capacity (primary muscle cells) (123, 404). It is not known whether this mutation or phenotype improves exercise endurance in humans. However, electromyography indices of rate of fatigue during a 60-s test of isometric contraction suggest that skeletal muscle of the carriers is less prone to fatigue (404). Studies in pigs may further define the role of the PRKAG3-activating mutation given similar physiology to humans. The activating PRKAG3 mutation (R200Q) was identified in pigs (124) and leads to muscle glycogen accumulation as well as elevated muscle glycolytic and oxidative capacity (405). So far, only short-term treadmill exercise tests (∼6–8 min) have been applied to the pig model, and no improvement in performance has been reported (406).

Although rodent models often display minor genetic variation compared with humans, the value of these models in relation to exercise performance and metabolism may be questioned. The endurance-like tests often applied are less physiologically relevant for the natural behavior of rodents, and indeed their metabolism is different in various aspects. Murine skeletal muscle has only 1/10 the amount of glycogen compared with human skeletal muscle, indicating a highly different whole-body glucose metabolism between and within organs. Furthermore, the role of muscle glycogen for exercise performance during treadmill exercise of mouse is still debated (407, 408). Nevertheless, introducing the activating PRKAG3 mutation in transgenic mice leads to a phenotype with elevated muscle glycogen levels, high oxidative capacity, and enhanced contractile performance in isolated glycolytic muscle *in vitro* (128, 409, 410). However, any performance improvement during swimming (410) or other forms of *in vivo* exercise has not been reported so far. Activating mutations in PRKAG1 (R70Q or H151R) have also been introduced in skeletal (predominantly glycolytic) muscle of mice (126, 127). These models also display glycogen accumulation, and at least 1 model was reported to have improved treadmill exercise endurance by a factor of 2 (126). Thus, the phenotype obtained by expression of an activating AMPK mutation in skeletal muscle would be predicted to promote *in vivo* exercise endurance, but the evidence to support such consequence is scarce.

The question rises as to whether AMPK activation by genetic or pharmacologic means alone or in combination with exercise may lead to improved exercise performance. If mice carrying the activating PRKAG3 mutation perform an acute bout of swimming exercise, the gene response is greater in the carriers compared with the control animals (411). However, whether this translates to superior functional adaptations at the protein/organelle level and improved exercise performance remains unknown. In fact, in pigs, differences and similarities in enzyme activities (*e.g.*, HK, CS, LDH) observed at baseline between carriers and noncarriers of the activating PRKAG3 mutation are maintained after exercise training, suggesting that adaptations to exercise training are not potentiated by chronic elevated AMPK activity (406, 412). Long-term exercise training studies with detailed phenotypic evaluation might provide insights into any synergistic adaptive response to exercise training when performed in the context of genetically elevated basal AMPK activity. Several pharmacological studies using various indirect and direct AMPK agonists support the notion that AMPK activation is sufficient to drive muscle adaptation toward high glycogen content, glycolytic and oxidative capacity, and mitochondrial biogenesis. Three studies have addressed whether this also associates with improved exercise performance (400, 413, 414). The study by Narkar *et al.* (400) demonstrated that 4 wk of AICAR treatment induced an increased exercise endurance phenotype during treadmill running. However, this study did not apply AMPK-deficient animal models to address whether AMPK in skeletal muscle was indeed needed for this adaption. Marcinko *et al*. (413) treated insulin-resistant mice fed HFD with R419 (Fig. 4) for 6 wk (mitochondrial complex 1 inhibitor) and observed an improvement in treadmill exercise capacity (413). This effect was dependent on AMPK β1 and β2 expression in skeletal muscle. Similarly, Um *et al*. (414) found that resveratrol increased treadmill exercise capacity in HFD-fed WT mice but not in whole-body AMPKα2- or AMPKα1-KO mice. Thus, in a likely exercise-intolerant model (due to HFD), pharmacological activation of AMPK in skeletal muscle improves exercise performance. From the perspective of physical activity in health, this is a promising observation. However, the available data do not offer solid scientific proof that AMPK activation (genetic or pharmacological) associates with improved exercise performance in metabolic healthy organisms or in well-trained athletes. On the other hand, data to disprove this claim are also not strong. Although the role of muscle AMPK is unclear, exercise training in combination with metformin treatment of obese prediabetic subjects (415) and insulin-resistant subjects (416) as well as exercise training in combination with resveratrol treatment of elderly persons (417) have been reported to induce similar or even less adaptive responses on factors such as *V*o_2peak_. Resveratrol treatment of animals in combination with exercise training has revealed improved adaptations compared with exercise alone (418, 419). This provides another example of the limited knowledge transfer between species. Based on the above-mentioned findings and the emergence of direct AMPK agonists that target β2-containing complexes in skeletal muscle (PF-739 and MK-8722), future studies should aim at determining the potential for such activators to improve exercise capacity in both healthy and diseased models.

By various mechanisms, AMPK has been proposed to inhibit the regulation of mTORC1 signaling and thus to inhibit skeletal muscle strength and hypertrophy. In accordance with this hypothesis, mTORC1 activation in muscle by strength training is lost or compromised when high-intensity sprint cycle exercise is performed before or immediately after strength training (420, 421), whereas moderate-intensity endurance exercise does not produce this effect (422, 423). Interestingly, AICAR-induced AMPK activation dismisses muscle mTOR signaling in response to high frequent electrical muscle stimulation in rats (424). Furthermore, in a model of muscle overload, the AMPKα1 complex is activated (425). In accordance with this finding, skeletal muscle growth after overload-induced muscle hypertrophy is enhanced in mice lacking the AMPKα1 isoform, which relates to elevated mTOR signaling (426). In agreement with this, knockout of the primary AMPKα2 upstream kinase LKB1 (425) and the AMPKγ3 isoform (427) does not affect overload-induced muscle hypertrophy. The idea that AMPKα1 is important in the interference between endurance and strength training is not straightforward, and studies of concurrent training should be performed with more detailed evaluation of AMPK heterotrimer complex activities to bring further insight to the AMPK–mTOR interaction. Although the rodent studies are in strong support for such interaction, it should not be forgotten that exercise regulates a range of other signals (428), some of which may act in such settings of interference (*e.g.*, PKA, MAPK, SIRTs).

## AMPK: A KEY PLAYER IN SKELETAL MUSCLE PLASTICITY, REGENERATION, AND REPAIR

Metabolic and nonmetabolic functions of AMPK, including its role in plasticity of skeletal muscle, have been investigated in tissues of various species (49). Skeletal muscle displays a remarkable ability to adapt its phenotype in terms of size, composition, and metabolic properties in response to environmental stimuli. Skeletal muscle fibers rapidly adapt to drastic changes in energy demands during exercise through fine-tuning of the balance between catabolic and anabolic processes (49).

Chronic *in vivo* activation of AMPK by AICAR administration inhibits overload-induced skeletal muscle hypertrophy (429), suggesting a crucial role of AMPK for the control of myofiber size. The α1 catalytic subunit of AMPK is activated by overload-induced hypertrophy (a supraphysiological muscle loading) (425, 426, 430). Therefore, even if AMPKγ3 seems to be dispensable for skeletal muscle hypertrophy induced by functional overload (427) and if the cross-section area of myofibers is reduced in skeletal muscle depleted of AMPK β1 and β2 (146, 431), it is not surprising that knocking out AMPKα1 results in greater overload-induced hypertrophy (426). Of note, the degree of muscle cell hypertrophy in response to activation of the protein synthesis pathway is lower in AMPKα2-deficient muscle cells (430). Furthermore, *in vitro* studies have demonstrated that AMPK negatively regulates hypertrophy of muscle cells (426, 430, 432, 433). Finally, AMPKα mdKO mice possess fibers with larger cross-sectional area as compared with WT mice, further supporting the notion that AMPKα negatively influences muscle growth (432). Because AMPK decreases mTORC1 activity *in vitro* through phosphorylation of TSC2 at Thr1345 (226) or raptor at Ser792 (229), these responses appear to be related to the deactivation of the signals involved in the protein synthesis pathway mTOR/p70S6K. Overload-induced hypertrophy results in the activation of molecular brakes that may feed back to attenuate the rate of skeletal muscle growth (434). Indeed, Hamilton *et al.* (434) have demonstrated that overload-induced stress results in the activation of AMPKα1 and phosphorylation of TSC2 Thr1345, which limits activation of mTORC1. Finally, knockout of AMPK induces myotube hypertrophy and up-regulation of p70S6K phosphorylation in primary cultured myoblasts under nonstimulated conditions (432). Taken together, these findings show that AMPK may act as a negative regulator of skeletal muscle hypertrophy through down-regulation of protein synthesis pathways.

Exercise training results in a more oxidative muscle phenotype that is likely mediated by an oxidative fiber type switch. Whether AMPK triggers an oxidative slow twitch MyHC phenotype has not been demonstrated convincingly. Thus, one study suggests that skeletal muscle of the PRKAG1-activating mutation model has a higher proportion of IIA fibers (285). Some observations suggest a MyHC IIB toward a IIA conversion in skeletal muscle of pigs carrying the PRKAG3 activating mutation (412, 435). However, such differences have not been reported in the transgenic mouse model (128) or in human carriers (123) of the PRKAG3 activating mutation. Reports on fiber type composition in muscle of other AMPK transgenic mouse models are also somewhat limited. One study suggests that lack of functional AMPKα2 leads to less MyHC type IIA/X–positive fibers (285), which is in part in line with observations of decreased MyHC IIX and increased MyHC IIb–positive fiber area in skeletal muscle of the LKB1-KO mouse model (436). However, another study found that the lack of both AMPKα1 and AMPKα2 leads to a muscle enriched in MyHC type I and perhaps also type IIA fibers (72). Studies of similar or other AMPK-deficient mouse models (AMPK kinase dead, β2M-KO, β1β2M-KO) report unchanged fiber type distribution (74, 146, 437). One study reports that lack of functional AMPKα2 attenuates exercise-induced muscle fiber type remodeling toward a distribution enriched in MyHC IIA/X fibers (285). Thus, genetic evidence for AMPK being an important mediator in promoting the expression of an oxidative slow-twitch MyHC phenotype is inconsistent. However, many of these studies are based on a limited number of observations and are often limited to a few muscle groups.

Besides its high adaptation capability, adult skeletal muscle possesses the capacity to regenerate after injury. Skeletal muscle regeneration requires the activation, migration, proliferation, and fusion of muscle stem cells to form new functional myofibers. The process of adult myogenesis requires the establishment of specific interactions of muscle cells with other cell types, including immune cells (438, 439). The inflammatory response during skeletal muscle regeneration is a spatially and temporally coordinated process. Whereas the first steps of the inflammatory response are associated with damage-associated macrophages that secrete proinflammatory cytokines, resolution of inflammation is associated with restorative macrophages that exhibit an anti-inflammatory phenotype and directly support myogenesis (440). The resolution of inflammation is a critical step, and its alteration impairs skeletal muscle regeneration (441, 442). Indeed, providing anti-inflammatory cues too early during the inflammatory phase or blocking anti-inflammatory cues after the first days of injury alters the regeneration process (443), showing that the temporal transition of proinflammatory to anti-inflammatory phases during skeletal muscle regeneration is crucial (444). In parallel to its role as an energy sensor, AMPK activation is associated with a decrease in the inflammatory response (445–447). Recent evidence has demonstrated that macrophage AMPKα1 is crucial for the resolution of inflammation during skeletal muscle regeneration, linking metabolism and inflammation (448). Indeed, specific deletion of AMPKα1 in macrophages induces a defect in macrophage skewing (*i.e.*, the transition from the damage-associated phenotype to the restorative macrophage phenotype) (449), triggering the impairment of skeletal muscle regeneration. During the regeneration process, AMPKα1 activation, dependent on phosphorylation by CaMKKβ but not by LKB1, is linked to the phagocytosis of apoptotic/necrotic muscle debris, triggering macrophage skewing (448).

AMPK is also involved in regulation of muscle stem cells (MuSCs) themselves, which, during regeneration, activate and recapitulate the myogenic program to repair damaged myofibers. MuSCs are capable of both differentiation to repair muscle tissue and self-renewal to replenish the stem cell pool, which is a crucial process to maintain the pool of MuSCs for further needs. Interestingly, LKB1 was shown to limit MuSC proliferation through the AMPK/mTORC1 pathway (450). The AMPK-regulated transcription factor FOXO3 enhances MuSC self-renewal *via* the activation of Notch signaling, an essential pathway for the maintenance of MuSC quiescence (451). In addition, regulation of stem cell fate seems to be dependent on the metabolic state of the cell (452, 453). In skeletal muscle, it has recently been shown that metabolic reprogramming promotes activation of MuSCs, identifying a tight link between the metabolic state of MuSCs and epigenetic modifications that regulate their myogenic commitment (454). In this context, AMPK controls muscle stem cell fate. AMPKα1-KO muscle stem cells displayed a high self-renewal rate and showed a Warburg-like switch of their metabolism to higher glycolysis through the regulation of the activity of LDH, a new functional target of AMPK in stem cells (455).

Contrary to healthy skeletal muscle, degenerating myopathies are characterized by permanent attempts of regeneration associated with persistent inflammation. Interestingly, animal models depleted for AMPK specifically in skeletal muscle fibers display a myopathic phenotype (72, 263, 431). Furthermore, chronic activation of AMPK by AICAR treatment of the dystrophic mdx mouse, a murine model for Duchenne myopathy, improves metabolic function and histologic pattern of the skeletal muscle. In this context, AMPK activation may not only target myofibers and mitochondrial metabolism but also inflammation in the myopathic muscle. Indeed, fibrosis, IL-6, and IL-10 levels are reduced in the muscle of AICAR-treated mdx mice (456). Deciphering the molecular pathways triggered by AMPK activation in myogenic (myofibers, MuSCs) and nonmyogenic cells (immune cells, fibroblastic cells, endothelial cells) will help to finely tune the potential benefits of activating this pathway to treat muscle disorders.

## AMPK: A THERAPEUTIC TARGET?

### AMPK and Duchenne muscular dystrophy

The inherited neuromuscular disorder Duchenne muscular dystrophy (DMD) causes life-limiting muscle wasting in boys and young men. The underlying etiology is a lack of functional dystrophin protein in muscle. Because no cure is available, ways to maintain muscular function are of significant interest to patients with DMD. Of particular interest for this review is the idea that AMPK activation might promote some aspects of the oxidative myogenic program and the observation that the slow oxidative myofiber phenotype is more resistant to dystrophin defects than the faster glycolytic myofiber phenotype (457).

By assembling with multiple partners, dystrophin provides a strong and elastic link between the intracellular cytoskeleton and the extracellular matrix and transmits mechanical and cellular signals (458). A lack of dystrophin leads to diminished sarcolemma integrity, mitochondria dysfunction, compromised calcium handling, myofibrillar protein degradation, apoptosis, and necrosis (459). Interestingly, a lack of both dystrophin and utrophin A (DmD^mdx^/Utrn KO) in the mouse mimics the human DMD disease better than a lack of dystrophin alone (460). Reintroducing utrophin A by overexpression in muscle of such models rescues the phenotype by decreasing fiber regeneration and by normalizing both dystrophin localization at the PM and expression of developmental proteins (461). Due to consequence similarities these proteins may have redundant roles within the cell, giving rise to this rescue effect of utrophin A. Utrophin A expression is calcineurin/nuclear factor of activated T cell–dependent and thus is part of the oxidative fiber programming (462). As discussed previously, AMPK may play an important role in inducing the oxidative myogenic program, and observations from several animal studies in which mdx mice have been treated with AMPK activating compounds such as AICAR, metformin, and resveratrol suggest that targeting AMPK in skeletal muscle may ameliorate the pathology observed in patients with DMD (12, 463, 464). Furthermore, studies suggest that up-regulation of utrophin A is key to the functional adaptations in the mdx mouse model after chronic AICAR treatment (465), although genetic evidence linking these effects to AMPK action is lacking. In fact, the idea is challenged by the apparent lack of effect on utrophin A gene expression in muscle after both acute and chronic treatment with the direct pan-AMPK activator PF-739 despite other indices of an up-regulated oxidative muscle fiber program (161). Nevertheless, endurance-type exercise regimes in animal models of DMD (mdx mice) induce adaptations toward remodeling of the muscle phenotype (466). In line with this finding, it has been suggested that patients with DMD may benefit from exercise training programs attempting to avoid disuse-induced muscle atrophy and dysfunction (467, 468). Whether activation of AMPK in skeletal muscle during exercise is necessary for these effects of exercise needs to be clarified.

### AMPK in obesity and diabetes

Obesity and related metabolic disorders such as type 2 diabetes and dyslipidemia are emerging as major global health challenges due to a complex interplay between obesity-favoring environmental factors, such as sedentary lifestyle, reduced physical activity levels, overconsumption of high-caloric food, and a permissive genetic prevalence (469, 470). Insulin resistance is one of the earliest hallmarks of the prediabetic state and arises from impaired glucose and fatty acid uptake and metabolism in a number of tissues, notably skeletal muscle, which is responsible for the majority of glucose disposal upon insulin stimulation and exercise. Given the importance of AMPK in the metabolic responses of skeletal muscle, a great deal of interest has focused on understanding AMPK signaling in skeletal muscle induced by either exercise or pharmacological activators as a treatment modality for type 2 diabetes. Thus, during the last decade there has been a major drive to develop small-molecule AMPK activators, and the identification of A769662 held promise in drug development for the treatment of T2D (156) until it was reported that A769662 predominantly activates AMPKβ1-containing complexes (70), which do not seem to exist in human skeletal muscle (69, 76). The importance of targeting the specific AMPK complexes in skeletal muscle has been highlighted by studies using compounds stimulating AMPK activity of both β1- and β2-containing complexes. It has been shown that the pan-AMPK activator 991 efficiently activates AMPK in isolated mouse skeletal muscle and elicits metabolic effects appropriate for treating T2D by stimulating glucose uptake and fatty acid oxidation (160, 471). The clinical relevance for the use of nonselective activators of all AMPK complexes to lower plasma glucose levels through increasing skeletal muscle glucose uptake has recently been demonstrated in rodents and nonhuman primates (161, 162). Thus, acute dosing of the pan-AMPK agonist PF-739 activated AMPK in skeletal muscle and liver and was capable of lowering blood glucose levels of diet-induced obese mice (161). Treatment with PF-739 resulted in increased glucose disposal rate but had no impact on endogenous glucose production (161). In addition, the blood glucose–lowering effect of PF-739 was diminished in mice deficient of AMPKα1 and AMPKα2 in skeletal muscle but not in liver, indicating that activation of AMPK in skeletal muscle is the appropriate means for the treatment of patients with type 2 diabetes independently of hepatic AMPK activation (161). Similarly, the pan-AMPK activator MK-8722, a potent activator of β2-containing complexes, enhanced glucose uptake in skeletal muscle *in vitro* and *in vivo* and improved glucose tolerance in diabetic rodents and nonhuman primates (162). However, although systemic pharmacological activation of AMPK by MK-8722 showed beneficial improvements of glucose homeostasis, potential cardiac safety concerns were reported. Long-term MK-8722 treatment was associated with reversible cardiac hypertrophy but without apparent adverse consequences (162). This effect could be reminiscent of the physiologic cardiac hypertrophy found in elite athletes due to cardiac AMPK activation during training (472).

Studies investigating AMPK activity in skeletal muscle of humans with obesity and type 2 diabetes have yielded mixed results. Some groups report decreased AMPK activity in skeletal muscle of obese people with or without type 2 diabetes compared with lean control subjects (321, 473), whereas others report intact regulation of AMPK activity and signaling both at rest and in response to exercise (120, 209, 474). Despite these observations, the contribution of reductions in muscle AMPK activity in the development of insulin resistance and obesity remains unclear. Indeed, knockout mouse models lacking AMPK specifically in skeletal muscle display comparable body mass, adiposity, glucose tolerance, and insulin sensitivity compared with control animals fed a control chow diet as well as a HFD (72, 74). These data indicate that loss of AMPK *per se* is not sufficient to induce HFD-induced obesity or insulin resistance. Nevertheless, there is substantial evidence suggesting that improvements in whole-body insulin sensitivity are associated with elevated AMPK activity. It is now well documented that physiologic (*e.g.*, exercise training) or pharmacological AMPK activation can combat insulin resistance and metabolic dysfunction caused by chronic nutrient excess (137, 475). Several studies have shown that pharmacological AMPK activation mimics the effects of regular exercise and calorie restriction and decreases the risk of progression of diabetes and the development of skeletal muscle insulin resistance (476–478). As such, a recent study using different genetically modified mouse models with altered AMPK activity has provided evidence to support that AMPK is essential for AICAR-induced elevation of muscle insulin sensitivity likely through phosphorylation of TBC1D4, a downstream target of insulin and AMPK signaling regulating GLUT4 translocation (129). In addition to its role in enhancing insulin-stimulated glucose uptake, repeated AICAR treatment has been reported to induce improvements in insulin-stimulated fatty acid uptake and oxidation (479). Although AICAR treatment represents a promising strategy to improve muscle insulin sensitivity in type 2 diabetes, observations in humans have so far been inconclusive (402, 403, 480, 481), given its relatively high threshold for AMPK activation in skeletal muscle (144, 480, 481). On the other hand, the reported impact of *in vivo* AICAR infusion on suppressing hepatic glucose output in patients with type 2 diabetes (481) and animal models (478, 482, 483) may provide interesting new leads for future pharmacological interventions. Finally, findings suggest that AMPK may suppress inflammatory signaling in skeletal muscle (484), which may contribute to its insulin-sensitizing action because it is well established that obesity-linked type 2 diabetes is associated with a low-grade chronic inflammatory state (485). Indeed, both AICAR and metformin have been shown to exert anti-inflammatory effects in many cell types (*e.g.*, myocytes, adipocytes, and macrophages) as well as in mature skeletal muscle and adipose tissue of LPS-challenged rats, a model of metabolic endotoxemia (486). Based on these observations and on findings of intact resting and exercise-induced activation of AMPK in skeletal muscle of patients with type 2 diabetes (120, 209, 474), this signifies a potential role of AMPK in skeletal muscle as a pharmacological target for prevention and treatment of inflammation and insulin resistance in various dysmetabolic conditions.

## CONCLUSIONS

AMPK has garnered considerable interest since its identification as an energy sensor and a critical regulator of cell metabolism in the 1980s. In skeletal muscle, numerous studies have highlighted the role of AMPK as a mediator of cell signaling pathways that are intrinsically linked to muscle function and metabolism (*i.e.*, glucose uptake, fatty acid oxidation, mitochondrial biogenesis, protein metabolism, and muscle plasticity). Although these observations are consistent with the idea that AMPK is essential for a healthy muscle phenotype, a lack of AMPK in skeletal muscle in general only leads to mild alterations in the muscle/whole-body phenotype. The simple explanation is that redundant mechanisms are put into play in the AMPK-deficient cell. However, another view could be that AMPK is dispensable under most physiologic situations, and only during severe energy perturbation (*e.g.*, exercise and starvation) does AMPK action become important for cell function and survival. In this view, skeletal muscle energy turnover at resting/normal conditions is very low, and the energy charge is only rarely challenged. Clearly, we need to dig deeper into the world of AMPK regulation and its role in physiology. For example, studies focusing on the regulation of the different heterotrimeric complexes, their specific roles, and the consequences of their subcellular localization would add a new dimension to the field. The observation of differential regulation of the heterotrimeric complexes opens up a largely unexplored avenue as to the downstream effectors of each of these and the mode of their regulation. The lack of proper and heterotrimer selective AMPK inhibitors/activators has to a large extent limited the investigation of the physiologic regulatory role of AMPK in muscle metabolism. Newer transgenic models of inducible knockout/overexpression may offer a way for such exploration. Because muscle tissue contributes ∼40% of the entire body mass, the general condition of this tissue is important for whole-body metabolism and overall health. Thus, being able to target AMPK specifically in skeletal muscle—perhaps through the unique expression of the AMPKγ3 complex—may offer an attractive opportunity, and further investigation is warranted to reveal the full potential for such strategies. More work is also needed to delineate the exact role(s) of AMPK in mature skeletal muscle. Given that exercise increases overall health as well as AMPK activity in skeletal muscle, we believe that finding ways of activating AMPK in skeletal muscle (preferably by exercise) may constitute an effective medical treatment for a number of diseases and may oppose the detrimental effects of age on daily physical capacity.

## Abbreviations

4E-BP1: 4E binding protein 1
ACC2: acetyl-CoA carboxylase 2
AICAR: 5-amino-1-β-d-ribofuranosyl-imidazole-4-carboxamide
BDNF: brain-derived neurotrophic factor
C2: 5-(5-hydroxyl-isoxazol-3-*yl*)-furan-2-phosphonic acid
CaMK: Ca^2+^/calmodulin-dependent protein kinase
CaMKKβ: Ca^2+^/calmodulin-dependent protein kinase kinase β
CD36: cluster of differentiation 36
DMD: Duchenne muscular dystrophy
ERR: estrogen-related receptor
FOXO3a: Forkhead box protein O3a
GBD: glycogen-binding domain
β-GPA: β-guanidinopropionic acid
GS: glycogen synthase
HDAC5: class II histone deacetylase 5
HFD: high-fat diet
KD: kinase domain
KO: knockout
LIF: leukemia inhibitory factor
LKB1: liver kinase B1
MAFbx: muscle atrophy F-box
mdKO: muscle-specific double knockout
MEF2: myocyte enhancer factor 2
mTORC1: mammalian target of rapamycin complex 1
MuRF1: muscle RING finger 1
MuSC: muscle stem cell
NAM: nicotinamide
NAMPT: nicotinamide phosphoribosyltransferase
NR4A: nuclear hormone receptor 4A
PDH: pyruvate dehydrogenase
PGC-1α: peroxisome proliferator–activated receptor γ coactivator 1α
PKD: protein kinase D
PLIN2: perilipin 2
ROS: reactive oxygen species
SIRT: NAD-dependent sirtuin
SNARK: sucrose nonfermenting AMPK-related kinase
TSC: tuberous sclerosis complex
ULK1: uncoordinated 51-like kinase 1
WADA: World Anti-Doping Agency
WT: wild type
ZMP: 5-aminoimidazole-4-carboxamide ribonucleotide

## AUTHOR CONTIBUTIONS

All authors contributed to the concept, design, and writing of this article and experimental work described in the text.
